# Evaluation of protein pattern changes in roots and leaves of *Zea mays *plants in response to nitrate availability by two-dimensional gel electrophoresis analysis

**DOI:** 10.1186/1471-2229-9-113

**Published:** 2009-08-23

**Authors:** Bhakti Prinsi, Alfredo S Negri, Paolo Pesaresi, Maurizio Cocucci, Luca Espen

**Affiliations:** 1 Dipartimento di Produzione Vegetale, University of Milan, via Celoria 2, I-20133 Milano, Italy; 2 Dipartimento di Produzione Vegetale, University of Milan c/o Fondazione Parco Tecnologico Padano, via Einstein – Località Cascina Codazza, I-26900 Lodi, Italy

## Abstract

**Background:**

Nitrogen nutrition is one of the major factors that limit growth and production of crop plants. It affects many processes, such as development, architecture, flowering, senescence and photosynthesis. Although the improvement in technologies for protein study and the widening of gene sequences have made possible the study of the plant proteomes, only limited information on proteome changes occurring in response to nitrogen amount are available up to now. In this work, two-dimensional gel electrophoresis (2-DE) has been used to investigate the protein changes induced by NO_3_^- ^concentration in both roots and leaves of maize (*Zea mays *L.) plants. Moreover, in order to better evaluate the proteomic results, some biochemical and physiological parameters were measured.

**Results:**

Through 2-DE analysis, 20 and 18 spots that significantly changed their amount at least two folds in response to nitrate addition to the growth medium of starved maize plants were found in roots and leaves, respectively. Most of these spots were identified by Liquid Chromatography Electrospray Ionization Tandem Mass Spectrometry (LC-ESI-MS/MS). In roots, many of these changes were referred to enzymes involved in nitrate assimilation and in metabolic pathways implicated in the balance of the energy and redox status of the cell, among which the pentose phosphate pathway. In leaves, most of the characterized proteins were related to regulation of photosynthesis. Moreover, the up-accumulation of lipoxygenase 10 indicated that the leaf response to a high availability of nitrate may also involve a modification in lipid metabolism.

Finally, this proteomic approach suggested that the nutritional status of the plant may affect two different post-translational modifications of phosphoenolpyruvate carboxylase (PEPCase) consisting in monoubiquitination and phosphorylation in roots and leaves, respectively.

**Conclusion:**

This work provides a first characterization of the proteome changes that occur in response to nitrate availability in leaves and roots of maize plants. According to previous studies, the work confirms the relationship between nitrogen and carbon metabolisms and it rises some intriguing questions, concerning the possible role of NO and lipoxygenase 10 in roots and leaves, respectively. Although further studies will be necessary, this proteomic analysis underlines the central role of post-translational events in modulating pivotal enzymes, such as PEPCase.

## Background

Under field conditions, nitrogen nutrition is one of the major factors that influence plant growth [1,2]. The availability of this nutrient affects many processes of the plant, among which development, architecture, flowering, senescence, photosynthesis and photosynthates allocation [1-7].

The low bio-availability of nitrogen in the pedosphere with respect to the request of the crops has spawned a dramatic increase in fertilization that has detrimental consequences on environment such as water eutrophication and increase in NH_3 _and N_2_O in the atmosphere [6,8].

Moreover, this side-effect is severe in the case of cereals, which account for 70% of food production worldwide. Indeed, in these crops the grain yield is strictly correlated with N supply but the use efficiency is not higher than 50% [9].

Because of the economical relevance, the feasibility to combine extensive physiological, agronomic and genetic studies as well as the high metabolic efficiency of C_4 _plants, maize (*Zea mays *L.) was proposed as the model species to study N nutrition in cereals [10].

Among nitrogen inorganic molecules, nitrate is the predominant form in agricultural soils, where it can reach concentrations three or more orders of magnitude higher than in natural soils [11,12].

In root cells, the uptake of this mineral nutrient involves inducible and constitutive transport systems [13]. Both systems mediate the transport of the anion by H^+ ^symport mechanisms [14-19] sustained by H^+^-ATPase [20-22].

The first step of nitrate assimilation, that occurs in both roots and shoots, involves its reduction to ammonia by nitrate reductase (NR) and nitrite reductase (NiR) enzymes, followed by transfer of ammonia to α-chetoglutaric acid by the action of glutamine synthetase (GS) and glutamate synthase (GOGAT) [23-25]. The pathway is induced in the presence of nitrate and shows many connections with other cellular traits, among which carbohydrate and amino acid metabolism, redox status and pH homeostasis [6,19,26,27]. Hence, nitrate and carbon metabolisms appear strictly linked and co- regulated, both locally and at long distance for the reciprocal root/leaf control, in response to the nutritional status of the plant and environmental *stimuli *[3,6,26-28].

In the last years, some transcriptomic analyses have been conducted to shed light on the molecular basis of these regulatory mechanisms. Wang and co-workers studied the transcriptomic changes occurring after exposure to low and high nitrate concentrations in whole plants of *Arabidopsis thaliana*, by means of microarray and RNA gel blot analysis [29]. Besides the genes already known to be regulated by the presence of nitrate, the authors found new candidate genes encoding for regulatory proteins such as a MYB transcription factor, a calcium antiporter, putative protein kinases and several metabolic enzymes. Another study conducted by Scheible and co-workers [7] reports a comparative transcriptomic analysis of *Arabidopsis thaliana *seedlings grown in sterile liquid culture under nitrogen-limiting and nitrogen-replete conditions by using Affymetrix ATH1 arrays and (RT)-PCR. The authors observed that the response to nitrogen availability involved a deep reprogramming of primary and secondary metabolisms. These data well describe the complexity of nitrogen pathway as well as the direct and/or indirect consequences that nitrogen availability exerts on the whole metabolism of the plant.

Starting from these results it should be now desirable to deepen the knowledge about the changes at translational and post-translational levels in response to nitrogen availability. In the last decade, the improvement in technologies for protein study and the widening of gene sequences made possible the study of the plant proteomes [30-34].

In this context, the availability of a large EST assembly and the efforts in sequencing maize genome [35] contributed to improve the use of maize, as highlighted by a large number of studies conducted on this species, among which the proteomic characterizations of leaf [36], of chloroplasts in bundle sheath and mesophyll cells [37] and of pericycle cells of primary roots [38].

At the present time, to the best of our knowledge no studies on nitrogen nutrition in maize were conducted by this approach. The only two proteomic works regarding this issue in cereals are based on the use of 2-DE to compare the leaves [39] and the roots [40] of two wheat varieties exposed to different levels of nitrogen. These works pointed out some significant differences, correlated to N availability during the plant growth, in the protein profiles of both organs.

In order to obtain further information, in this work we investigated protein accumulation changes induced by nitrate in both roots and leaves of *Zea mays *plants. The attention was focused on the changes in the pattern of protein soluble fractions caused by the addition of 10 mM nitrate to the hydroponic solution, after a period in which the plants were grown in the absence of nitrogen. Firstly, the changes of some biochemical parameters were measured to describe the physiological response occurring after nitrate addition and were used to define the sampling time for proteomic analysis. These experiments led to compare the proteomes of plants previously grown for 17 days in absence of nitrogen and incubated for further 30 h without the nutrient or in the presence of 10 mM nitrate. Through 2-DE and LC-ESI-MS/MS analyses a first characterization of the proteome changes occurring in maize plants in response to an increase in nitrate availability was obtained. The results show how many of these changes were related to enzymes of the nitrate assimilation or metabolic pathways strictly linked to it (*e.g*. pentose phosphate pathway and photosynthesis), but also reveal new proteins that may play a role in the nitrate responses.

## Results and discussion

### Experimental design and biochemical parameters

The aim of this work was to apply a proteomic approach to study the changes in protein patterns of root and leaf organs of maize plants in the first phase of exposure to high availability of nitrate, comparable to agricultural conditions, after a growth period under nitrogen starvation. This is a typical condition in which the addition of nitrate induces an increase in uptake and assimilation of this nutrient [5,28].

**Figure 1:**
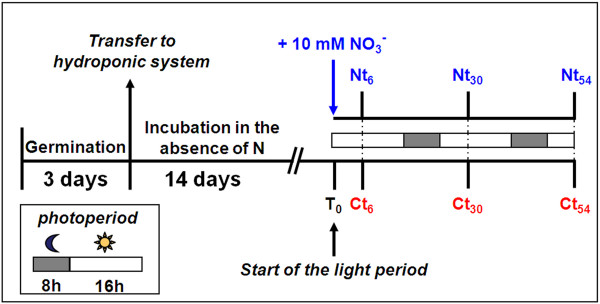
**Experimental design**. *Zea mays *seeds were germinated in the dark. After 3 days, the seedlings were transferred in a hydroponic system and grown for 14 days in the absence of nitrogen (T_0_), afterwards the plants were incubated for further 54 h in the same condition (Control, C) or in the presence of 10 mM KNO_3 _(N). For details see the methods section.

The need for a simultaneous analysis of the root and the leaf organs of starved plants, with completely developed but not stressed leaf apparatus, led to the definition of the experimental design showed in Figure 1. Briefly, seedlings were transferred into a hydroponic system after 3 days of germination and grown for further 14 days in a solution deprived of nitrogen. After that, at the beginning of the light period (T_0_), some plants were maintained in the same nutritional condition (control, C) whereas others were transferred in a nutrient solution containing 10 mM NO_3_^- ^(N). In order to define the sampling time for proteomic analysis, the changes of biochemical parameters in response to NO_3_^- ^were firstly evaluated. Roots and leaves were collected at T_0 _time and after 6, 30 and 54 h of nitrate exposure.

At these sampling times, the plants achieved the developmental stage corresponding to the complete expansion of the third leaf (pictures of harvested plants are showed in Additional file 1). The qualitative comparison between the C and N plants revealed some morphological differences. In particular, while the plants appeared very similar at the T_0 _sampling time, after 30 h the expansion of the fourth leaf was slightly more evident in N plants with respect to the C ones. This trend was more pronounced at 54 h and, only in C plants, was accompanied by the comparison of faint yellow areas in the leaf blades. In the tested conditions, no significant differences were observed in root system.

**Figure 2:**
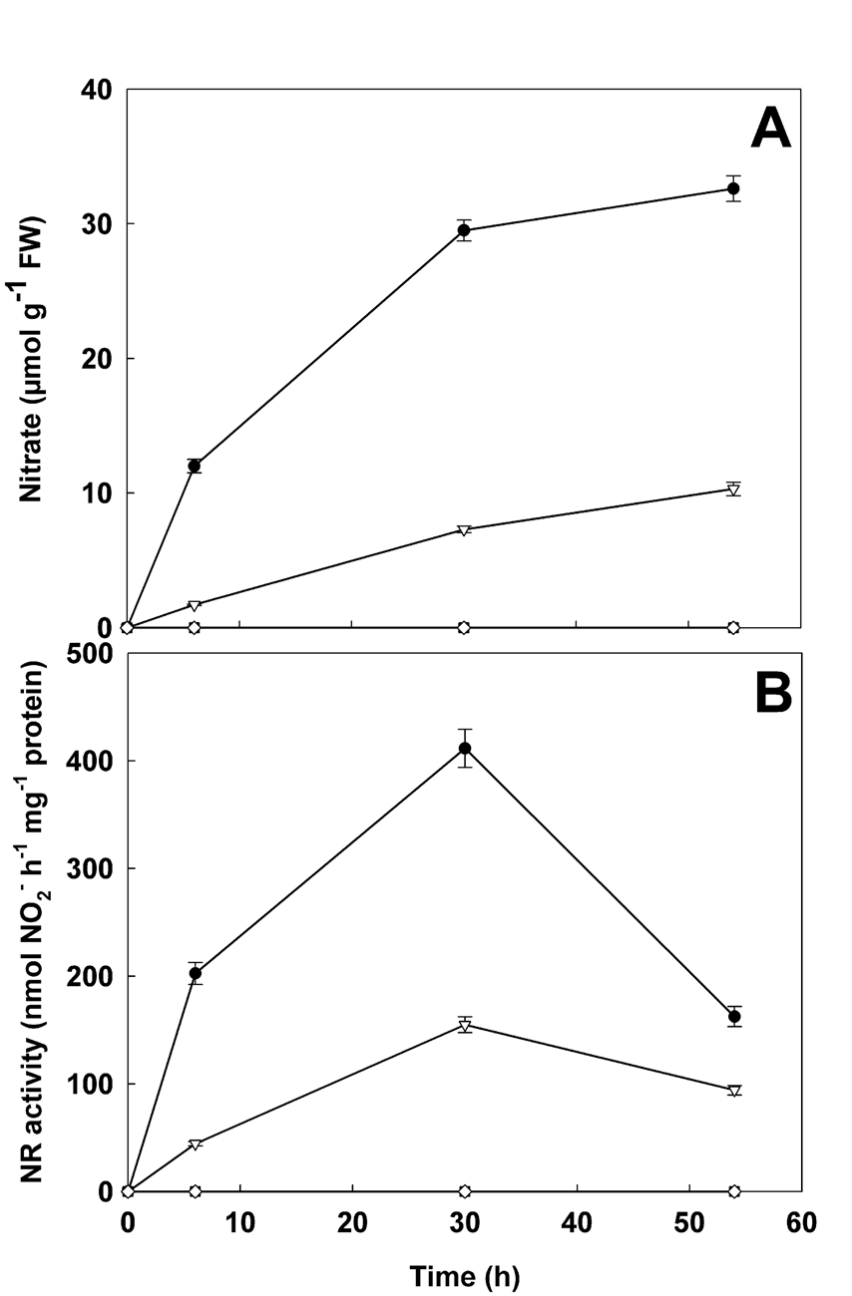
**Nitrate content and nitrate reductase activity**. Time course of the changes in nitrate content (A) and nitrate reductase activity (B) in roots (close circles and closed squares) and leaves (open triangle and open rhombuses) of *Zea mays *plants, previously grown for 17 days under nitrogen starvation (T_0_) and incubated for further 6, 30 and 54 h in the absence (closed squares and open rhombuses) or in the presence (closed circles and open triangles) of 10 mM NO_3_^-^. In roots and leaves of starved plants, both nitrate and NR activity were undetectable. Values are the mean ± SE of three independent biological samples analyzed in triplicate (n = 9).

**Figure 3:**
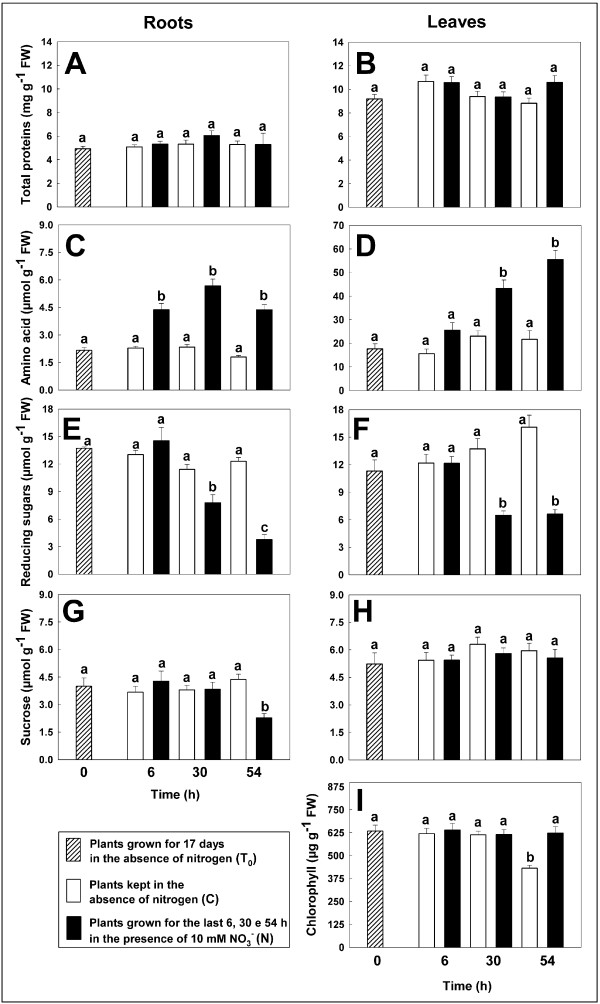
**Total proteins, amino acids, reducing sugars, sucrose and chlorophyll content**. Time course of the changes in the content of total proteins, amino acids, reducing sugars and sucrose in roots (A, C, E and G) and leaves (B, D, F, and H) and chlorophyll content in leaves (I) of *Zea mays *plants, previously grown in the absence of nitrogen for 17 days (T_0_) and incubated for further 6, 30 and 54 h in the absence (C) or presence of 10 mM NO_3_^- ^(N). Values are the mean ± SE of three independent biological samples analyzed in triplicate (n = 9). Samples indicated with the same letters do not differ significantly according to Tukey's test (*p *< 0.01).

In order to characterize the physiological status of the plants, the changes in nitrate content and NR activity (Figure 2) as well as the levels of proteins, amino acids, reducing sugars, sucrose and chlorophyll were evaluated (Figure 3).

In roots and leaves of starved plants, both nitrate and NR activity were undetectable. After the addition of the nutrient to hydroponic solution the levels of nitrate progressively increased in plant tissues, reaching a level of 32.6 and 10.3 μmol of NO_3_^- ^g^-1 ^FW after 54 h in roots and leaves respectively (Figure 2A). A parallel dramatic increase of NR activity was measured until the 30^th ^h of NO_3_^- ^exposure, while at the longest time considered (54 h) a decreased activity was observed (Figure 2B). This trend was more evident in the roots in which a more rapid and large availability of nitrate took place. The total protein levels did not change significantly in all the conditions tested (Figure 3A and 3B), while a sharp increase in free amino acids was detected in both organs after nitrate addition (Figure 3C and 3D). Moreover, the levels of amino acids were higher in the leaves than in the roots. Although many factors are involved in the overall amino acid levels, these results may suggest a contribution of translocation of nitrogen compounds between the two organs. Nitrate exposure also induced a decrease in reducing sugars in both organs (Figure 3E and Figure 3F), while only in the roots of the plants exposed for 54 h to 10 mM NO_3_^- ^a drop of sucrose took place (Figure 3G).

Taken together, these results well describe the induction trend of NO_3_^- ^assimilation pathway, as suggested by the increase of NR activity and amino acids accompanied by the consequent decrease of reducing sugars, the main source of carbon skeletons [41]. In roots, where photosynthesis cannot satisfy this request and/or the demand of carbon skeleton is high, sucrose pool was also affected. The changes in carbohydrate availability and the increase of amino acid levels also explain the decrease in NR activity observed in roots at the 54^th ^h. In fact, these data are in agreement with the inhibitory effect on NR evocated by an increase of some amino acids, mainly asparagine and glutamine [5,42]. Moreover, it is know that NR activity increases after sucrose addition whilst the low sugar content, condition that we observed in the roots of N plants, affects the nitrate reduction system [5,42,43]. The results suggested that this feedback mechanism was activated in roots of the plants exposed for 54 h to 10 mM NO_3_^-^. Finally, only at the 54^th ^h, a significant decrease in chlorophyll content (Figure 3I) was measured in the leaves of starved plants, thus suggesting that the first symptoms of stress were appearing.

### 2-DE analysis and protein identification

The biochemical and physiological data showed that the plants incubated for the last 30 h in the presence of 10 mM NO_3_^- ^were in a condition in which nitrogen metabolism is completely activated in both root and leaf organs and that, at the same time, no stress symptoms were detectable in the control plants. Starting from these results, the proteomic study was conducted by analyzing the soluble protein fractions extracted from roots and leaves of plants incubated for the last 30 h in the absence or in the presence of 10 mM NO_3_^-^.

The ratio between dry and fresh weight as well as the total protein content appeared similar both in the roots and in the leaves of C and N samples (Table 1). The adopted protocol permitted to obtain an extraction yield of soluble proteins of about 14% and 20% for roots and leaves, respectively. Moreover, no significant differences were observed between C and N plants.

**Table 1:** Evaluation of the procedure for the extraction of soluble proteins from roots and leaves of plants grown in the two conditions compared in the proteomic analysis.

**Organ**	**Condition**	**FW/DW**	**Total proteins (mg g^-1^FW)**	**Extraction yield of soluble proteins (%)**
**Root**	**C plants**	**8.63 **± 0.05	**5.32 **± 0.34	**13.39 **± 0.72
	**N plants**	**8.43 **± 0.34	**6.04 **± 0.41	**14.63 **± 0.69
				
**Leaf**	**C plants**	**8.45 **± 0.09	**9.39 **± 0.45	**19.17 **± 0.99
	**N plants**	**8.64 **± 0.15	**9.35 **± 0.43	**20.96 **± 0.43

In the table, the fresh/dry weight (FW/DW), the content of total protein (mg g ^-1^FW) and the % yield of the extraction of soluble proteins (% of extracted soluble proteins respect to the total content) for the roots and the leaves of the plants compared by proteomic analysis are reported. The fresh weight of the roots was 0.56 ± 0.03 and 0.60 ± 0.04 g in C and N plants, respectively. The fresh weight of the leaves was 0.79 ± 0.03 and 0.86 ± 0.04 g in C and N plants, respectively.

C plants: plants kept in the absence of nitrogen; N plants: plants grown for the last 30 h in the presence of 10 mM NO_3_^-^. Values are the mean ± SE of three independent biological samples analyzed in triplicate (n = 9).

**Figure 4:**
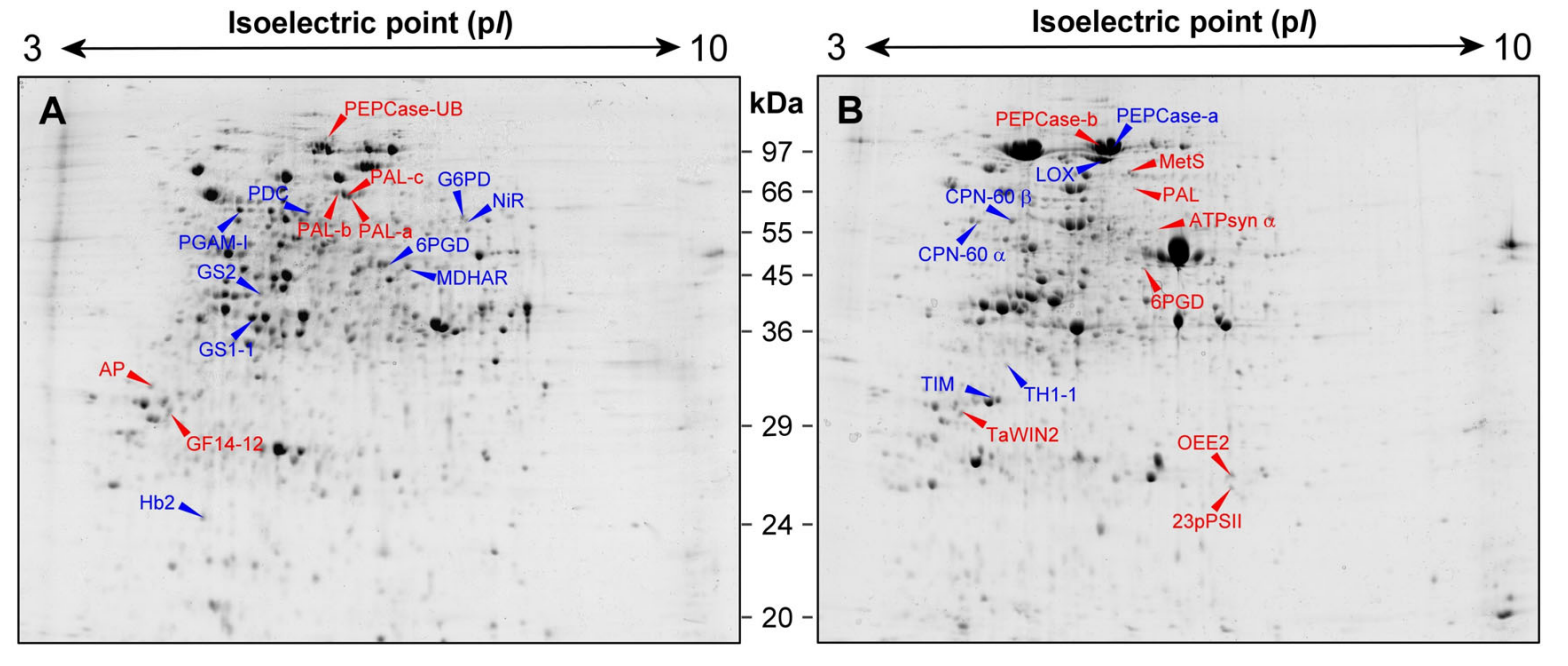
**2-DE maps**. Representative 2-DE maps of soluble protein fractions extracted from roots (A) and leaves (B) of *Zea mays *plants. Proteins (400 μg) were analyzed by IEF at pH 3–10, followed by 12.5% SDS-PAGE and visualized by cCBB-staining. Name abbreviations, corresponding to those in Tables 2 and 3, indicate the spots, identified by LC-ESI-MS/MS, showing significant changes of at least two-fold in their relative volumes (t-test, *p *< 0.05) after the exposure to 10 mM nitrate for 30 h. Proteins that increased or decreased after this treatment are reported in blue or in red, respectively.

The 2-DE representative gels of the soluble fractions of root and leaf samples are shown in Figure 4. The electrophoretic analyses detected about 1100 and 1300 spots in roots and leaves gels, respectively. To ascertain the quantitative changes in the proteomic maps, the relative spot volumes (*%Vol*) were evaluated by software-assisted analysis. The Student's t-test (*p *< 0.05), coupled with a threshold of two-fold change in the amount, revealed that 20 spots in roots and 18 spots in leaves were affected by nitrogen availability.

The analysis of these spots by LC-ESI-MS/MS allowed to identify 15 and 14 proteins in root and leaf patterns, respectively. These proteins and the changes in their accumulation are shown in Tables 2 and 3, while further information of mass spectrometry (MS) analysis are reported in the Additional files 2 and 3.

**Table 2:** List of the spots identified in the roots and their change in abundance after the exposure to 10 mM nitrate for 30 h.

**Spot ID**	**Accession number**	**Protein description**	**Abbr.^*a*^**	***M*_*r*_^*b*^/*pI*^*b*^**	***M*_*r*_^*c*^/*pI*^*c*^**	**Change in level** [Relative volume (%)]	
						**Control**	**10 mM NO**_3_
**Glycolysis, gluconeogenesis, C-compound and carbohydrate metabolism**							
53	BAA28170	Phosphoenolpyruvate carboxylase	**PEPCase-UB**	*115.4/5.7*	*109.4/5.7*	**0.223 **± 0.022	**0.084 **± 0.032
	P69319	Ubiquitin			*8.5/6.6*		
216	P30792	2,3-bisphosphoglycerate-independent phosphoglycerate mutase	**PGAM-I**	*63.0/5.1*	*60.6/5.3*	**0.124 **± 0.086	**0.245 **± 0.011
231	AAL99745	Pyruvate decarboxylase	**PDC**	*62.4/5.5*	*65.0/5.7*	**0.080 **± 0.043	**0.167 **± 0.024
392	EAZ18378	6-phosphogluconate dehydrogenase^*d*^	**6PGD**	*50.1/6.1*	*50.1/5.5*	**0.080 **± 0.031	**0.275 **± 0.033
1162	NP_196815	Glucose-6-phosphate 1-dehydrogenase	**G6PD**	*60.3/6.7*	*67.2/8.5*	**0.002 **± 0.001	**0.010 **± 0.014
**Nitrogen metabolism, amino acid metabolism and protein/peptide degradation**							
268	ACG29734	Ferredoxin-nitrite reductase	**NiR**	*59.7/6.7*	*66.2/6.5*	**0.035 **± 0.054	**0.124 **± 0.084
483	P25462	Glutamine synthetase, chloroplastic	**GS2**	*42.2/5.2*	*41.0/5.4*^*e*^	**0.066 **± 0.015	**0.137 **± 0.059
538	P38559	Glutamine synthetase root isozyme 1	**GS1-1**	*38.7/5.1*	*39.2/5.6*	**0.210 **± 0.010	**0.480 **± 0.039
707	BAA06876	Aspartic protease	**AP**	*31.6/4.6*	*54.1/5.1*	**0.051 **± 0.043	**0.015 **± 0.065
**Secondary metabolism**							
171	AAL40137	Phenylalanine ammonia-lyase	**PAL-a**	*68.6/5.9*	*74.9/6.5*	**0.476 **± 0.034	**0.184 **± 0.012
172	AAL40137	Phenylalanine ammonia-lyase	**PAL-b**	*68.6/5.8*	*74.9/6.5*	**0.904 **± 0.136	**0.277 **± 0.026
1160	AAL40137	Phenylalanine ammonia-lyase	**PAL-c**	*68.0/5.8*	*74.9/6.5*	**0.713 **± 0.103	**0.275 **± 0.034
**Cell rescue, defense and virulence**							
390	NP_001061002	Putative monodehydroascorbate reductase ^*d*^	**MDHAR**	*50.1/6.2*	*52.8/6.8*	**0.127 **± 0.016	**0.275 **± 0.033
960	AAZ98790	hemoglobin 2	**Hb2**	*24.8/4.9*	*20.6/5.0*	**0.018 **± 0.061	**0.099 **± 0.068
**Unknown**							
774	Q01526	14-3-3-like protein GF14-12	**GF14-12**	*29.6/4.6*	*29.6/4.7*	**0.345 **± 0.034	**0.146 **± 0.028

Statistical information about LC-ESI-MS/MS analysis are reported in Additional files 2 and 3. Changes in the relative spot volumes are the mean ± SE of six 2-DE gels derived from three independent biological samples analyzed in duplicate (n = 6). Proteins were classified according to MIPS funcat categories.

^*a*^: Protein abbreviation

^*b*^: Experimental molecular weight (kDa) or isoelectric point

^*c*^: Theoretical molecular weight (kDa) or isoelectric point

^*d*^: Information obtained by alignment of the sequence through BLAST analysis against NCBI nr database

^*e*^: Values referred to the mature form of the protein

**Table 3:** List of the spots identified in the leaves and their change in abundance after the exposure to 10 mM nitrate for 30 h.

**Spot ID**	**Accession number**	**Protein description**	**Abbr.^*a*^**	***M*_*r*_^*b*^/*pI*^*b*^**	***M*_*r*_^*c*^/*pI*^*c*^**	**Change in level** [Relative volume (%)]	
						**Control**	**10 mM NO_3_**
**Nitrogen and amino acid metabolism**							
1094	BAB11740	TaWIN2	TaWIN2	*29.9/4.7*	*28.7/4.8*	**0.182 **± 0.009	**0.090 **± 0.014
254	AAL73979	Methionine synthase protein	MetS	*83.4/5.9*	*83.8/5.9*	**0.148 **± 0.020	**0.073 **± 0.008
**C-compound and carbohydrate metabolism**							
650	AAC27703	Putative cytosolic 6-phosphogluconate dehydrogenase	6PGD	*47.4/6.0*	*52.9/6.2*	**0.088 **± 0.005	**0.043 **± 0.007
**Photosynthesis**							
134	P04711	Phosphoenolpyruvate carboxylase 1	PEPCase-a	*104.4/5.8*	*109.3/5.8*	**0.990 **± 0.083	**2.770 **± 0.295
138	P04711	Phosphoenolpyruvate carboxylase 1	PEPCase-b	*104.4/5.7*	*109.3/5.8*	**2.220 **± 0.278	**1.090 **± 0.205
500	P05022	ATP synthase subunit alpha, chloroplastic	ATPsyn α	*55.9/6.1*	*55.7/5.9*	**0.042 **± 0.007	**0.015 **± 0.003
1065	NP_001063777	Putative triosephosphate isomerase, chloroplast precursor ^*d*^	TIM	*31.0/4.9*	*32.4/7.0*	**0.028 **± 0.009	**0.088 **± 0.014
1244	Q00434	Oxygen-evolving enhancer protein 2, chloroplast precursor	OEE2	*26.6/6.5*	*27.3/8.8*	**0.201 **± 0.013	**0.090 **± 0.011
1612	BAA08564	23 kDa polypeptide of photosystem II	23pPSII	*26.3/6.5*	*27.0/9.5*	**0.147 **± 0.008	**0.055 **± 0.006
**Protein folding and stabilization**							
462	NP_001056601	RuBisCO subunit binding-protein beta subunit ^*d*^	CPN-60 β	*58.5/5.1*	*64.1/5.6*	**0.079 **± 0.014	**0.164 **± 0.015
467	AAP44754	Putative rubisco subunit binding-protein alpha subunit precursor	CPN-60 α	*58.2/4.8*	*61.4/5.4*	**0.046 **± 0.004	**0.096 **± 0.004
**Metabolism of vitamins, cofactors, and prosthetic groups**							
999	Q41738	Thiazole biosynthetic enzyme 1-1, chloroplast precursor	TH1-1	*33.0/5.1*	*32.8/4.9*^*e*^	**0.010 **± 0.001	**0.048 **± 0.003
**Secondary metabolism**							
313	AAL40137	Phenylalanine ammonia-lyase	PAL	*70.2/6.0*	*74.9/6.5*	**0.076 **± 0.008	**0.023 **± 0.002
**Lipid metabolism**							
219	ABC59693	Lipoxygenase	LOX	*94.6/5.8*	*102.1/6.1*	**0.023 **± 0.011	**0.149 **± 0.011

Statistical information about LC-ESI-MS/MS analysis are reported in Additional files 2 and 3. Changes in the relative spot volumes are the mean ± SE of six 2-DE gels derived from three independent biological samples analyzed in duplicate (n = 6). Proteins were classified according to MIPS funcat categories.

^*a*^: Protein abbreviation

^*b*^: Experimental molecular weight (kDa) or isoelectric point

^*c*^: Theoretical molecular weight (kDa) or isoelectric point

^*d*^: Information obtained by alignment of the sequence through BLAST analysis against NCBI nr database

^*e*^: Values referred to the mature form of the protein

### Functional role and quantitative change of the proteins identified in roots

Many of the spots identified in roots were enzymes involved in nitrogen and carbon metabolisms (Table 2). According to the induction of the NO_3_^- ^assimilation pathway, in the roots of the plants incubated for the last 30 h in the presence of the nutrient, we observed an increase in the accumulation of nitrite reductase (spot 268, NiR) and of glutamine synthetase plastidial isoform (spot 483, GS2).

Moreover, in response to the demand of carbon skeletons and NADPH, which is used in non-green tissues for ferredoxin reduction [44], an increase in the levels of phosphoglycerate mutase (spot 216, PGAM-1), glucose-6-phosphate dehydrogenase (spot 1162, G6PD) and 6-phospho-gluconate dehydrogenase (spot 392, 6PGD) took place. These results well agree with previous array data that describe the responses to nitrate exposure in Arabidopsis and tomato [7,29,45].

An increase in accumulation of the cytosolic isoform of glutamine synthetase (spot 538, GS1-1) was also detected in roots of N plants. On the basis of identified peptides by MS analysis it was possible to discriminate among the 5 GS1 isoforms known in *Zea mays *(SwissProt reviewed database) and to restrict the possible identification to 2 of them (GS1-1 [Swiss-Prot:P38559] and GS1-5 [Swiss-Prot:P38563] [46]). The fact that Li and co-workers [46], through a Northern blot hybridization analysis, found that the transcript of *GS1-1 *gene was the only one expressed in roots, conducted to the specific identification of GS1-1 protein. Moreover, Sakakibara and co-workers [47] showed that *GS1-1 *transcript was the only induced by NO_3_^-^. The proteomic approach used in the present work allows to confirm these results at the translational level, demonstrating that in maize roots a cytosolic ammonia assimilation pathway can be activated also in response to nitrate.

Other spots that were found to increase their relative volumes in response to nitrate were a non-symbiotic hemoglobin and a monodehydroascorbate reductase (spot 960, Hb2 and spot 390, MDHAR). In a previous work on Arabidopsis, it was found that NO_3_^- ^induced *AtHB1 *and *AtHB2*, two genes that encode for non-symbiotic hemoglobins [7,29]. Scheible and co-workers [7] suggested that these proteins could change their abundance in relation to the redox status, whereas Wang and co-workers [29] speculated on the possibility that the induction of hemoglobin could aim at reducing oxygen concentration during NR synthesis, since molybdenium can be sensitive to oxygen. Besides, hemoglobin and MDHAR are known to be involved in the scavenging of NO that can be produced by cytosolic and/or plasmamembrane nitrate reductase when nitrite is used as substrate [48,49]. NO is a signaling molecule which is involved in many biochemical and physiological processes [50]. It has been reported that in plant roots, NO plays a role in growth, development and in some responses to environmental conditions, such as hypoxia [51]. Recently, a possible involvement of NO in the mediation of nitrate-dependent root growth in maize has been suggested [52]. According to this work, that describes a reduction of endogenous NO at high external NO_3_^- ^concentration, the observed concomitant up-accumulation of Hb2 and MDHAR in our experimental condition supports the hypothesis that they might contribute in controlling NO levels in root tissues after exposure to NO_3_^- ^[48,49,52].

The last protein found to be present in higher amount in N plants was a pyruvate decarboxylase (spot 231, PDC). This enzyme catalyzes the decarboxylation of pyruvic acid into acetaldehyde, the first step of the alcoholic fermentation. In particular, we identified the PDC isoenzyme 3 that has been previously found to be induced in hypoxia condition [53]. Although further studies are required to understand why PDC is induced by NO_3_^-^, we can observe that fermentation pathways are induced in response to redox status changes and that this condition could be also linked to the activation of the Hb/NO cycle (see above) [49,54].

Among the spots identified in roots, six showed a down-accumulation in N plants (Table 2). Three of them were identified as phenylalanine ammonia-lyase (spots 171, 172 and 1160, PAL-a, PAL-b and PAL-c). The MS analysis indicated for all three spots the same protein [GenBank:AAL40137] while the electrophoretic data showed some differences in M_r _and p*I*, suggesting that post-translational modification events may have occurred. It has been shown as low nitrogen availability induces transcripts encoding enzymes of phenylpropanoid and flavonoid metabolism, such as PAL, chalcone synthase and 4-coumarate:coenzyme A ligase, whilst after nitrogen repletion these activities are down-regulated [7,55]. Our proteomic data appear to be in agreement with these studies.

Previously, it was found that under low nitrogen availability four proteases (*e.g*. serine, aspartate/metalloproteases and two cysteine proteases) increased their activity to degrade non-essential proteins in order to remobilize this nutrient [56]. In this work, we found an aspartic protease belonging to the A1 family (spot 707, AP) that was down-regulated after NO_3_^- ^exposure. Moreover, the experimental M_r _appeared lower with respect to that expected for this protein, thus suggesting that this spot is referable to the active form of the enzyme [57]. These data support a new possible role for A1 protease family [57,58].

Phosphoenolpyruvate carboxylase activity is known to increase during nitrate assimilation, having a role in cell pH homeostasis and an anaplerotic function [14,19,59-61]. In addition, the monoubiquitination of this enzyme was recently well described in germinating castor oil seeds by Uhrig and co-workers [62]. It was found that this event is non-destructive and that this reversible post-translational modification of the enzyme reduces its affinity for PEP and its sensitivity to allosteric activators and inhibitors. The MS analysis of spot 53 (for sequence details see Additional file 4) identified 8 peptides, 7 of which matched with a PEPCase [DDBJ:BAA28170] (theoretical M_r_/p*I *equal to 109.4/5.7), while the last peptide belonged to an ubiquitin (UB) [Swiss-Prot:P69319] (theoretical M_r_/p*I *equal to 8.5/6.6). The experimental M_r _and p*I *of spot 53, that were 115.4 and 5.7 respectively, were in agreement with the monoubiquitination of the PEPCase (PEPCase-UB, theoretical M_r_/p*I *equal to 117.9/5.8 respectively). Moreover, the domain responsible to bind ubiquitin previously identified in PEPCase of other vascular plants is present in this maize PEPCase [62]. These results suggest that in maize roots the modulation of PEPCase activity in response to nitrogen availability could occur also through reversible monoubiquitination.

The last spot identified in roots that was down-regulated by NO_3_^- ^was the 14-3-3-like protein GF14-12 (spot 774, GF14-12). Previously, it was found that this protein is localized in the nucleus where it binds the DNA at the G-box regions in association with transcription factors and that it is involved in the regulation of gene expression [63,64]. More recently, it was described an interaction of 14-3-3 proteins with some transcription factors such as VP1, EmBP1, TBP and TFIIB [65]. Further studies are required to clarify the effective role of GF14-12, for which the functional information are still lacking.

### Functional role and quantitative change of the proteins identified in leaves

Many of the spots identified in leaves by LC-ESI-MS/MS analysis were proteins linked to the NO_3_^- ^assimilation as well as to the photosynthetic activity (Table 3).

The activity of NR can be modulated also at post-translational level through a phosphorylation event followed by binding of inhibitory 14-3-3 protein [66,67]. One of the spots analyzed in the leaves was identified as TaWIN2 (Table 3, spot 1094, TaWIN2), that was previously described to be involved in the NR inactivation [67]. We found that the level of this protein decreased in leaves of N plants, where NR activity was induced (Table 3).

According to the well known relationships existing between nitrogen and carbon metabolism, the changes in accumulation of some spots after NO_3_^- ^addition are consistent with an increase of photosynthesis rate. Two spots that raise after NO_3_^- ^addition were identified as CPN-60α and CPN-60β (spot 467 and 462, CPN-60α and CPN-60β, respectively), that are chaperonin proteins involved in folding of ribulose-1,5-bisphosphate carboxylase [68]. Moreover, a chloroplastic triosephosphate isomerase was up-regulated by NO_3_^- ^(spot 1065, TIM), while a cytosolic 6-phosphogluconate dehydrogenase (spot 650, 6PGD) was down-regulated, as expected when the request of reducing power could be satisfied by the increase in photosynthetic activity [69].

Spot 500 was identified as the α subunit of the chloroplastic ATP synthase (ATPsyn α), but unexpectedly it was more abundant in leaves of C plants. Although only a speculative interpretation of this result can be made, we could hypothesize that in leaves of the N plants ATP synthase should be activated and this process requires the reconstitution of the enzymatic complex in the thylakoid membranes [70]. Hence, to clarify this point, it should be necessary to investigate if the decrease of ATPsyn α observed in the soluble fraction of N plants is effectively accompanied by an increase of this protein in the membrane fraction.

Thiamine (*i.e*. vitamin B_1_) is required in many pathways, such as the Calvin cycle, the branched-chain amino acid pathway and pigment biosynthesis [71]. Along with higher request of this vitamin in leaves of N plants, where the activation of these pathways could take place, we identified, among the spots up-regulated by N, the thiazole biosynthetic enzyme (spot 999, TH1-1) that is known to be involved in thiamine biosynthesis [71].

Two spots were identified as PEPCase (spot 134 and 138, PEPCase-a and PEPCase-b respectively). In C_4 _plants such as maize, this enzyme plays a central role in photosynthesis, because it catalyses the primary fixation of atmospheric CO_2 _[72]. The catalytic activity and sensitivity of this enzyme are mediated by a reversible phosphorylation [73]. The experimental p*I*s of the spots 134 and 138 were 5.8 and 5.7, respectively. Moreover, these two PEPCase forms showed opposite changes in abundance in the leaves of plants grown in the last 30 h in the presence of NO_3_^- ^with respect to the controls. The results obtained in our work suggest that the two spots of PEPCase are referable to the phosphorylated (spot 138) and to the unphosphorylated (spot 134) form with a predicted p*I *of 5.7 and 5.8, respectively, that are known to correspond to the more and less active states of this enzyme [74]. Interestingly, despite the fact that data suggest an increase in the photosynthetic activity, the phosphorylated form was more abundant in the proteomic map of C plants. These results support the immunological observation by Ueno and co-workers [73] that the diurnal regulation of phosphorylation state of PEPCase appears delayed in nitrogen-limited conditions, suggesting that the circadian control of PEPcase is affected by nitrogen starvation.

Two of the spots down-regulated in leaves of N plants were a phenylalanine ammonia-lyase (spot 313, PAL) and a methionine synthase (spot 254, MetS). The decrease of PAL, observed also in root tissue (see above), is a further evidence that phenylpropanoid and flavonoid metabolisms are affected by nitrogen availability [7,55]. On the other hand, the change in accumulation of MetS is contrasting with a recent proteomic study performed on wheat by Bahrman and co-workers [39]. These authors found that the induction of this enzyme was positively related to nitrogen availability. This discrepancy could be associated to different genetic traits of the two species, as well as it could be linked to different experimental approaches adopted in the two studies. Nevertheless, it should be observed that in both these works a single spot referable to MetS was detected, while further information on total level and/or on activity of this enzyme is necessary to clarify this point.

The spot 219, which considerably increased in N plants, was a lipoxygenase (LOX). In particular, the analysis of the MS spectra identified the LOX codified by *ZmLOX10 *gene, which was found to be a plastidic *type 2 *linoleate 13-LOX [75]. The expression analysis of this gene revealed that its transcript was abundant in leaves and was regulated by a circadian rhythm with a trend strictly linked to the photosynthetic activity. Moreover, it has been proposed that ZmLOX10 is involved in the hydroperoxide lyase-mediated production of C_6_-aldehydes and alcohols and not in the biosynthesis of JA [75]. Although some evidences suggest a role of ZmLOX10 in the responses to (a)biotic stresses, its involvement in the diurnal lipid metabolism was also proposed [75,76].

At the same time, we identified two proteins as an oxygen-evolving enhancer protein 2 (spot 1244, OEE2) and a 23 kDa polypeptide of photosystem II (spot 1612, 23pPSII), which were down-accumulated in leaves of N plants (Table 3). Both have been classified as members of PsbP family that is one of the three extrinsic protein families composing the oxygen-evolving complex (OEC) of photosystem II in higher plants [77-79]. In addition, it was recently demonstrated that PsbP proteins are essential for the normal function of PSII and play a crucial role in stabilizing the Mn cluster *in vivo *[80]. Moreover, the stability of this class of protein seems related to the lipid composition of chloroplastic membranes that is also affected by nitrogen availability [81,82].

**Figure 5:**
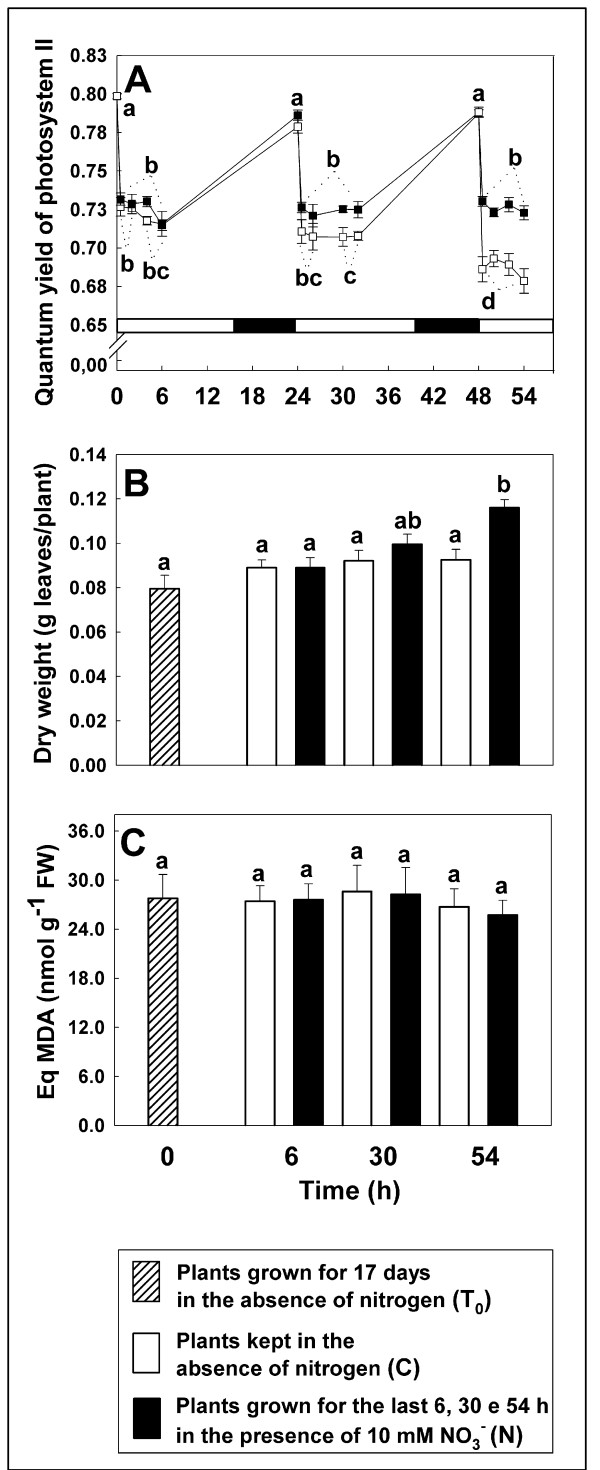
***F*_V_/*F*_M_, Φ_II_, dry weight and MDA in leaves**. Time course of the changes in *F*_V_/*F*_M _and Φ_II _(A), dry weight in leaves (B) and MDA levels (C) of *Zea mays *plants, previously grown for 17 days under nitrogen starvation (T_0_) and incubated for further 6, 30 and 54 h in the absence or presence of 10 mM NO_3_^-^. Symbol in Figure A: open squares, Control; closed squares, 10 mM NO_3_- ; horizontal bar: white bars, light periods; black bars, dark periods (for details see Figure 1). Values are the mean ± SE of three independent biological samples analyzed in triplicate (n = 9). Samples indicated with the same letters do not differ significantly according to Tukey's test (*p *< 0.01).

In order to elucidate the physiological meaning of these variations and to verify if they could be related to a stress status or to an alteration in photosynthetic performance, changes of both maximum quantum yield of photosystem II (*F*_V_/*F*_M_; dark adapted plants) and effective quantum yield of photosystem II (Φ_II_; light adapted plants), dry weight and MDA levels of shoot were measured (Figure 5). Although the *F*_V_/*F*_M _parameter, measured on over-night dark adapted plants at time points 0, 24 and 48 hours, resulted in very similar values between C and N plants (about 0.80; see also Figure 5A), the Φ_II _values showed a very slight decrease in C plants during the second period of illumination (C plants Φ_II_, 0.71 versus N plants, 0.73) and the difference became more marked between 48 and 54 hours of nitrogen starvation. Similar data could be obtained by monitoring biomass production at the different time points (Figure 5B), indicating that photosynthetic performances are highly impaired in C plants after 48–54 hours of treatment. Nevertheless, no changes in MDA were detected in all the conditions tested (Figure 5C).

Taken together these results indicate that at the 30^th ^h, the time point chosen for proteomic analysis, plants start feeling the different nitrogen content in the growth media without developing major stress symptoms and the associated pleiotropic effects.

These data sustain the hypothesis that ZmLOX10 could be involved in lipid metabolism of the chloroplast that is strictly depending on photosynthetic activity [75,76]. Further analyses are needed to unravel this possible intriguing role of ZmLOX10.

Considering the PsbP proteins, the change in accumulation of OEE2 and 23pPSII could indicate that OEC stability is affected by the N availability. Through time-course experiments, it will be possible to better correlate the relationship among N nutritional status, lipid metabolism, PsbP protein levels and PSII functionality.

## Conclusion

Many of the proteins found to change in accumulation in response to NO_3_^- ^were directly involved in the assimilation of this mineral nutrient. Moreover, the results underline the strict relationship between nitrogen and carbon metabolisms. The experimental design chosen for this proteomic study allows to emphasize some intriguing metabolic activities in both organs. Besides a dramatic increase of NO_3_^- ^assimilation pathway, the exposure to a high NO_3_^- ^concentration after a starvation period seems to induce a modification in NO metabolism in roots, that could depend on the need of responding to the new nutritional status. In leaves, many proteins were found to be (in)directly involved in the photosynthesis reactivation and in the maintenance of the chloroplastic functionality.

In addition, this proteomic analysis confirms the modulation by phosphorylation of the PEPCase in the leaves, suggesting that nitrogen availability could affect the circadian rhythms, as well as it shows that the form of this enzyme operating in roots could be modulated by monoubiquitination. Although further efforts are required to elucidate these results, the present study underlines the central role of post-translational events to modulate pivotal enzymes in plant metabolic response to NO_3_^-^.

## Methods

### Plant material and growth conditions

Maize (*Zea mays *L.) seeds of T250 inbred line, kindly provided by Prof. Zeno Varanini of Udine University – Italy, were germinated in the dark at 26°C on blotting paper saturated with deionized water. After 72 h, seedlings were transferred to a hydroponic system placed in a growth chamber with a day/night regime of 16/8 h and a PPFD of 200 μmol m^-2 ^s^-1 ^at plant level, with a temperature of 22°C in the dark and 26°C in the light and with a relative humidity of 70%. Seedlings were grown using of the following solutions: (i) 4 mM CaSO_4 _for the first 48 h; (ii) 0.4 mM CaSO_4_, 0.2 mM K_2_SO_4_, 0.175 mM KH_2_PO_4_, 0.1 mM MgSO_4_, 5 μM KCl, 20 μM Fe-EDTA, 2.5 μM H_3_BO_3_, 0.2 μM MnSO_4_, 0.05 μM CuSO_4_, 0.2 μM ZnSO_4_, 0.05 μM Na_2_MoO_4 _(growing solution) for the following 12 days. After this 17 days-long period of N starvation (T_0_), plants were transferred in a fresh growing solution added (N) or not (C) with 10 mM KNO_3_. The pH of all the growth solutions was adjusted to 6.1 and the solutions were changed every three days. All hydroponic solutions were continuously aerated by an electric pump.

At T_0 _stage and after a period of 6, 30 and 54 h plants were harvested, washed with distilled water and then blotted with paper towels. Finally, roots and leaves were separated and the samples were frozen in liquid N_2 _and stored at -80°C. The roots used for determining nitrate content were rinsed twice in ice-cold 0.4 mM CaSO_4 _solution for 15 min for removing the anion present in the apoplast before sampling.

### Levels of nitrate

Nitrate was extracted from the tissues by homogenizing the samples previously boiled in 4 volumes of distilled water for 15 min. The homogenate was centrifuged at 12,000 *g *for 20 min to obtain a clarified supernatant. Nitrate content was measured by adding 0.8 ml of 5% (w/v) salicylic acid in concentrated sulfuric acid solution to 0.2 ml of the supernatant. The mixture was stirred vigorously and allowed to react over 20 min, afterwards 19 ml of 2 N NaOH were slowly added and the resulting colour was read at 410 nm [83].

### Nitrate reductase activity

NR was extracted by using 4 volumes of ice-cold 50 mM pH 7.8 MOPS-KOH buffer containing 5 mM EDTA, 5 mM NaF, 2 mM MSH, 1 mM PMSF, 10 μM FAD, 1 μM leupeptin and 10 μM chymostatin. The homogenates were centrifuged at 13,000 *g *for 15 min at 4°C. NR activity was measured as described by Ferrario-Méry *et al*. [84] using a reaction mixture consisting of 50 mM pH 7.5 MOPS-KOH buffer, 1 mM NaF, 10 mM KNO_3_, 0.17 mM NADH, 10 mM MgCl_2 _and 5 mM EDTA. The reaction was blocked after 10 or 20 min by adding an equal volume of sulphanilamide (1%, w/v in 3 M HCl) followed by n-naphtylethylethylenediamine dihydrochloride (0.02%, w/v). 30 min later, the concentration of NO_2_^- ^was determined spectrophotometrically at 540 nm. The protein concentration was determined by 2-D Quant Kit (GE Healthcare).

### Determination of reducing sugars, sucrose, amino acids, total proteins and chlorophyll

Reducing sugars, sucrose and amino acids were extracted by homogenizing frozen tissues in 4 volumes of ice-cold 0.5 M perchloric acid (PCA). The homogenate was centrifuged for 10 min at 13,000 *g *at 4°C and the resulting pellet was washed with the same volume of PCA and then centrifuged again in the same conditions. KOH was added to the collected supernatant (to pH 7.6) to remove excess PCA. Reducing sugars were measured according to the colorimetric method by Nelson [85]. Total soluble sugars were determined by the same method boiling an aliquot of PCA extract for 1 h before neutralization. Sucrose was estimated from the difference between total soluble and reducing sugars. Total amino acids were measured by the ninhydrin method [86].

Total proteins were extracted as previously described by Martínez and co-workers [87] by homogenizing the samples, previously powdered in liquid nitrogen, in 4 volumes of a 125 mM pH 8.8 Tris-HCl buffer containing 1% (w/v) SDS, 10% (w/v) glycerol, 50 mM Na_2_S_2_O_5_. The homogenate was centrifuged at 13,000 *g *for 20 min to obtain a clarified supernatant. The protein content was measured by using 2-D Quant Kit (GE Healthcare).

Chlorophyll was extracted by homogenizing the leaves, previously powdered in liquid nitrogen, in 4 volumes of 80% pre-cooled acetone (v/v). The homogenate was centrifuged at 13,000 g for 20 min at 4°C to obtain a clarified supernatant. Chlorophyll concentration was measured according to Lichtenthaler [88].

### Determination of malondialdehyde and chlorophyll fluorescence of the leaves

Malondialdehyde (MDA) was assayed by the method of Heath & Packer [89]. Frozen samples were homogenized with 4 volumes of ice-cold 0.1% (w/v) trichloroacetic acid (TCA) and centrifuged at 13,000 *g *for 20 min at 4°C. An equal volume of 20% (w/v) TCA plus 0.5% (w/v) thiobarbituric acid was added to the supernatants, which were subsequently heated at 95°C for 30 min. The extracts were then clarified by centrifugation at 13,000 *g *for 10 min, and the difference between the absorbance at 532 and 600 nm was measured. The MDA equivalent was calculated from the resulting difference using the extinction coefficient of 155 mM^-1 ^cm^-1^.

In order to determine the photosynthetic performance, the chlorophyll fluorescence was measured by using a portable continuous-excitation type fluorometer (Handy-PEA, Hansatech Instrument). The maximum quantum efficiency of photosystem II (*F*_V_/*F*_M_) was calculated on over-night dark adapted plants, according to the equation (*F*_M_-*F*_0_)/*F*_M_, where *F*_0 _and *F*_M _are the fluorescence levels when plastoquinone electron acceptor pool (Qa) is fully oxidized and transiently fully reduced, respectively [90]. The photosynthetic performance of light adapted plants was evaluated by monitoring the effective quantum yield of photosystem II (Φ_II_) defined as (*F*_M_'-*F*_0_')/*F*_M_' [91], where *F*_M_'and *F*_0_' represent the maximal and minimal fluorescence emission of photosystem II under light conditions.

### Statistical analyses of biochemical and physiological measurements

For all the biochemical and physiological measurements, the experimental design consisted in three independent biological samples each analyzed in triplicate (n = 9).

One-way analysis of variance (ANOVA) followed by the *post hoc *Tukey test (*p *< 0.01) was used to verify the significance of the variations measured among all the tested parameters. This statistical analysis was performed using the software STATISTICA 7.

### Extraction of protein samples for 2-DE analysis

Three independent biological replicates were extracted for each condition. Frozen samples, each composed by leaves or roots of 6 plants, were finely powdered in liquid nitrogen using a pestle and mortar, added with PVPP [0.5% and 1% (w/w) for roots and leaves samples, respectively], homogenized in 4 volumes of extraction buffer [0.5 M Tris-HCl pH 8, 0.7 M sucrose, 10 mM EDTA, 1 mM PMSF, 1 μM leupeptin, 0.1 mg mL^-1 ^Pefabloc (Fluka), 0.2% (v/v) MSH] and centrifuged at 13,000 *g *at 4°C for 20 min. The resultant supernatant was centrifuged at 100,000 *g *at 4°C for 38 min to obtain the soluble fraction. Proteins were then purified using the method previously described by Hurkman and Tanaka [92] by adding an equal volume of ice-cold Tris buffered phenol (pH 8) to the supernatant. Samples were shaken for 30 min at 4°C, incubated for 2 h at 4°C and finally centrifuged at 5,000 *g *for 20 min at 4°C to separate the phases. Proteins, grouped in the upper phenol phase, were precipitated by the addition of five volumes of -20°C pre-cooled 0.1 M ammonium acetate in methanol and the incubation at -20°C overnight. Precipitated proteins were recovered by centrifuging at 13,000 *g *at 4°C for 30 min and then washed again with cold methanolic ammonium acetate and three times with cold 80% (v/v) acetone. The final pellet was dried under vacuum and dissolved in IEF buffer [7 M urea, 2 M thiourea, 3% (w/v) CHAPS, 1% (v/v) octylphenoxy polyethoxy ethanol (NP-40), 50 mg mL^-1 ^DTT and 2% (v/v) IPG Buffer pH 3–10 (GE Healthcare)] by vortexing and incubating for 1 h at room temperature. Samples were centrifuged at 10,000 *g *for 10 min and the supernatants stored at -80°C until further use. Protein concentration was determined by 2-D Quant Kit (GE Healthcare).

### 2-DE analysis

Protein samples (400 μg) were loaded on pH 3–10, 24 cm IPG strips passively rehydrated overnight in 7 M urea, 2 M thiourea, 3% (w/v) CHAPS, 1% (v/v) NP-40, 10 mg mL^-1 ^DTT and 0.5% (v/v) IPG Buffer pH 3–10. IEF was performed at 20°C with current limit of 50 μA/strip for about 90 kVh in an Ettan IPGphor (GE Healthcare). After IEF, strips were equilibrated by gentle stirring for 15 min in an equilibration buffer [100 mM Tris-HCl pH 6.8, 7 M urea, 2 M thiourea, 30% (w/v) glycerol, 2% (w/v) SDS] added with 0.5% (w/v) DTT for disulfide bridges reduction and for an additional 15 min in the same equilibration buffer to which 0.002% (w/v) bromophenol blue and 4.5% w/v iodoacetamide for cysteine alkylation were added. Second-dimensional SDS-PAGE [93] was run in 12.5% acrylamide gels using the ETTAN DALT *six *apparatus (GE Healthcare). Running was first conducted at 5 W/gel for 30 min followed by 15 W/gel until the bromophenol blue line ran off. Two replicates were produced for each biological replicate, thus obtaining six gels *per *condition (n = 6).

Proteins were stained using the colloidal Coomassie Brilliant Blue G-250 (cCBB) procedure, as previously described by Neuhoff and co-workers [94]. The gels were scanned in an Epson Expression 1680 Pro Scanner and analyzed with ImageMaster 2-D Platinum Software (GE Healthcare). Automatic matching was complemented by manual matching. The molecular weights of the spots were deduced on the basis of the migration of SigmaMarkers™ wide range (MW 6.500 – 205.000), while p*I*s were determined according to the strip manufacturer's instructions (GE Healthcare) reporting on the reference gel of the software-assisted analysis the values of p*I *predicted for any given length of the strip. Both M_r _and p*I *of the spots of interest were then determined by using software-automated algorithm.

Relative spot volumes (*%Vol*) of the six replicate gels *per *condition were compared and were analyzed according to the Student's t-test to verify whether the changes were statistically significant (*p *< 0.05). This analysis was performed by using SigmaStat software. Only spots showing at least a two-fold change in their relative volumes were considered for successive analyses.

### Protein in-gel digestion and LC-ESI-MS/MS analysis

Spots excised from gels stained with cCBB were digested as described by Magni and co-workers [95] with some refinements. In detail, after the destaining procedure, spots were dried under vacuum on a centrifugal evaporator and incubated in 10 mM DTT, 100 mM NH_4_HCO_3 _for 45 min at 56°C. The solution was replaced with 55 mM iodoacetamide, 100 mM NH_4_HCO_3 _and the spots were incubated for 30 min in the dark at room temperature. After that, spots were briefly washed with 100 mM NH_4_HCO_3 _and again incubated for 15 min in 50% (v/v) acetonitrile (ACN), for 3 min in 100% ACN, for 3 min in 100 mM NH_4_HCO_3_, for 15 min in 50 mM NH_4_HCO_3 _in 50% (v/v) ACN and finally dried under vacuum. The following phases consisting in the protein digestion with trypsin [Sequencing grade modified Trypsin V5111, Promega, Madison] and in the recovery of peptides were carried out as described in the article above cited.

The LC-ESI-MS/MS experiments were conducted using a Surveyor (MS pump Plus) HPLC system directly connected to the ESI source of a Finnigan LCQ DECA XP MAX ion trap mass spectrometer (ThermoFisher Scientific Inc., Waltham, USA). Chromatography separations were obtained on a BioBasic C18 column (180 μm I.D × 150 mm length, 5 μm particle size), using a linear gradient from 5% to 80% solvent B [solvent A: 0.1% (v/v) formic acid; solvent B: ACN containing 0.1% (v/v) formic acid] with a flow of 2.5 μl/min. ESI was performed in positive ionization mode with spray voltage and capillary temperature set at 3 kV and at 220°C, respectively. Data were collected in the full-scan and data dependent MS/MS mode with collision energy of 35% and a dynamic exclusion window of 3 min.

Spectra were searched by TurboSEQUEST^® ^incorporated in BioworksBrowser 3.2 software (ThermoFisher Scientific Inc., Waltham, USA) against the *Zea mays *protein subset, *Zea mays *EST subset and against the protein NCBI-nr database, all downloaded from the National Center for Biotechnology Information [96]. The searches were carried out assuming parent ion and fragment ion mass tolerance of ± 2 Da and ± 1 Da, respectively, two possible missed cleavages *per *peptide, fixed carboxyamidomethylation of cysteine and variable methionine oxidation. Positive hits were filtered on the basis of peptides scores [Xcorr ≥ 1.5 (+1 charge state), ≥ 2.0 (+2 charge state), ≥ 2.5 (≥ 3 charge state), ΔCn ≥ 0.1, peptide probability < 1 × 10^-3 ^and Sf ≥ 0.70] [97]. If needed, identified peptides were used in protein similarity search performed by alignment analyses against the NCBI-nr database using the FASTS algorithm [98]. Physical properties of the characterized proteins were predicted by *in silico *tools at ExPASy [99].

## Authors' contributions

BP contributed to the conception of the experimental design, carried out the determination of biochemical and physiological parameters, protein extraction, 2-DE, protein characterization by LC-ESI-MS/MS and analyzed the MS data, participated in writing the methods section of the manuscript. ASN analyzed the gels and performed statistical analyses. PP measured fluorescence parameters. MC contributed to the interpretation of the results and took part in the critical revision of the manuscript. LE conceived the study, coordinated the experiments, participated to the determination of biochemical and physiological parameters, wrote and edited the manuscript. All authors read and approved the final manuscript.

## Supplementary Material

Additional file 1 **Pictures of the plants**. File shows the pictures of the experimental plant material at the different sampling times. Click here for file

Additional file 2 **Caption of Additional file **3. Caption and legend of Additional file 3. Click here for file

Additional file 3 **Data on protein identification by LC-ESI-MS/MS and bioinformatic analysis**. Table shows the sequence of the peptides identified by MS/MS and the statistical information related to peptides, proteins and alignment analyses Click here for file

Additional file 4 **Details of the protein sequences assigned to spot 53**. File shows in detail the sequences of the PEPCase and UB proteins that were identified analyzing the spot 53 by LC-ESI-MS/MS, as well as the sequence alignment analysis to verify the presence of the domain involved in monoubiquitination of the enzyme. Click here for file

## References

[B1] Marschner H (1995). Mineral Nutrition of Higher Plants.

[B2] Barker AV, Bryson GM, Barker AV, Pilbeam DJ (2007). Nitrogen. Handbook of Plant nutrition.

[B3] Stitt M (1999). Nitrate regulation of metabolism and growth. Curr Opin Plant Biol.

[B4] Brouquisse R, Masclaux C, Feller U, Raymond P, Lea PJ, Morot-Gaudry JF (2001). Protein hydrolysis and nitrogen remobilisation in plant life and senescence. Plant Nitrogen.

[B5] Stitt M, Müller C, Matt P, Gibon Y, Carillo P, Morcuende R, Sheible WR, Krapp A (2002). Steps towards an integrated view of nitrogen metabolism. J Exp Bot.

[B6] Miller AJ, Cramer MD (2004). Root nitrogen acquisition and assimilation. Plant Soil.

[B7] Scheible WR, Morcuende R, Czechowski T, Fritz C, Osuna D, Palacios-Rojas N, Schindelasch D, Thimm O, Udvardi MK, Stitt M (2004). Genome-wide programming of primary and secondary metabolism, protein synthesis, cellular growth processes, and the regulatory infrastructure of Arabidopsis in response to nitrogen. Plant Physiol.

[B8] Jackson LE, Burger M, Cavagnaro TR (2008). Roots, nitrogen transformation and ecosystem services. Annu Rev Plant Biol.

[B9] Lawlor DW, Lemaire G, Gastal F, Lea PJ, Morot-Gaudry JF (2001). Nitrogen, plant growth and crop yield. Plant Nitrogen.

[B10] Hirel B, Bertin P, Quillere I, Bourdoncle W, Attagnant C, Dellay C, Gouy A, Cadiou S, Retailliau C, Falque M, Gallais A (2001). Towards a better understanding of the genetic and physiological basis for nitrogen use efficiency in maize. Plant Physiol.

[B11] Hagedorn F, Bucher JB, Schleppi P (2001). Contrasting dynamics of dissolved inorganic and organic nitrogen in soil and surface waters of forested catchments with Gleysols. Geoderma.

[B12] Owen AG, Jones DL (2001). Competition for amino acids between wheat roots and rhizosphere microorganisms and the role of amino acids in plant N acquisition. Soil Biol Biochem.

[B13] Orsel M, Filleur S, Fraisier V, Daniel-Vedele F (2002). Nitrate transport in Plants: which gene and which control?. J Exp Bot.

[B14] Ullrich CI, Novacky AJ (1990). Extra and intracellular pH and membrane potential changes by K^+^, Cl^-^, H_2_PO_4 _and NO_3 _uptake and fusicoccin in root hairs of *Limnobium stoloniferum *. Plant Physiol.

[B15] McClure PR, Kochian LV, Spanwick RM, Shaff JE (1990). Evidence for cotransport of nitrate and protons in maize roots. I. Effects of nitrate on the membrane potential. Plant Physiol.

[B16] Meharg AA, Blatt MR (1995). NO_3_^- ^transport across the plasma membrane of *Arabidopsis thaliana *root hairs: kinetic control by pH and membrane voltage. J Membrane Biol.

[B17] Crawford NM, Glass ADM (1998). Molecular and physiological aspects of nitrate uptake in plants. Trends Plant Sci.

[B18] Huang NC, Liu KH, Lo HJ, Tsay YF (1999). Cloning and functional characterization of an Arabidopsis nitrate transporter gene that encodes a constitutive component of low-affinity uptake. Plant Cell.

[B19] Espen L, Nocito FF, Cocucci M (2004). Effect of NO_3_^- ^transport and reduction on intracellular pH: an *in vivo *NMR study in maize roots. J Exp Bot.

[B20] Palmgren MG (2001). Plant plasma membrane H^+^-ATPases: powerhouses for nutrient uptake. Annu Rev Plant Physiol Plant Mol Biol.

[B21] Santi S, Locci G, Monte R, Pinton R, Varanini Z (2003). Induction of nitrate uptake in maize roots: expression of putative high affinity nitrate transporter and plasma membrane H^+^-ATPase isoforms. J Exp Bot.

[B22] Sondergaard TE, Schulz A, Palmgren MG (2004). Energization of transport processes in plants. roles of the plasma membrane H1-ATPase. Plant Physiol.

[B23] Oaks A, Hirel B (1985). Nitrogen metabolism in roots. Annu Rev Plant Physiol.

[B24] Meyer C, Stitt M, Lea PJ, Morot-Gaudry JF (2001). Nitrate reduction and signalling. Plant Nitrogen.

[B25] Hirel B, Lea PJ, Lea PJ, Morot-Gaudry JF (2001). Ammonia assimilation. Plant Nitrogen.

[B26] Crawford NM (1995). Nitrate: nutrient and signal for plant growth. Plant Cell.

[B27] Paul MJ, Foyer CH (2001). Sink regulation of photosynthesis. J Exp Bot.

[B28] Forde BG (2002). Local and long-range signalling pathways regulating plant responses to nitrate. Annu Rev Plant Biol.

[B29] Wang R, Guegler K, LaBrie ST, Crawford NM (2000). Genomic analysis of a nutrient response in Arabidopsis reveals diverse expression patterns and novel metabolic and potential regulatory genes induced by nitrate. Plant Cell.

[B30] Rossignol M (2001). Analysis of the plant proteome. Curr Opin Biotech.

[B31] Roberts JKM (2002). Proteomics and future generation of plant molecular biologists. Plant Mol Biol.

[B32] Yarmush ML, Jayaraman A (2002). Advances in proteomic technologies. Annu Rev Biomed Eng.

[B33] Patterson SD, Aebersold RH (2003). Proteomics: the first decade and beyond. Nat Genet Suppl.

[B34] Jorrín-Novo JV, Maldonato AM, EchevarríaZomeòo S, Valledor L, Castllejo MA, Curto M, Valero J, Sghaier B, Donoso G, Redonado I (2009). Plant Proteomics update (2007–2008): Second-generation proteomic techniques, an appropriate experimental design, and data analysis to fulfil MIAPE standards, increase plant proteome coverage and expand biological knowledge. J Proteomics.

[B35] Lawrence CJ, Dong Q, Mary L, Polacco ML, Seigfried TE, Brendel V (2004). MaizeGDB, the community database for maize genetics and genomics. Nucleic Acids Res.

[B36] Porubleva L, Velden KV, Kothari S, David J, Oliver DJ, Parag R, Chitnis PR (2001). The proteome of maize leaves: Use of gene sequences and expressed sequence tag data for identification of proteins with peptide mass Fingerprints. Electrophoresis.

[B37] Majeran W, Cai Y, Sun Q, van Wijk KJ (2005). Functional differentiation of bundle sheath and mesophyll maize chloroplasts determined by comparative proteomics. Plant Cell.

[B38] Dembinsky D, Woll K, Saleem M, Liu Y, Fu Y, Borsuk LA, Lamkemeyer T, Fladerer C, Madlung J, Barbazuk B, Nordheim A, Nettleton D, Schnable PS, Hochholdinger F (2007). Transcriptomic and proteomic analyses of pericycle cells of the maize primary root. Plant Physiol.

[B39] Bahrman N, Le Gouls J, Negroni L, Amilhat L, Leroy P, Laìné AL, Jaminon O (2004). Differential protein expression assessed by two-dimensional gel electrophoresis for two wheat varieties grown at four nitrogen levels. Proteomics.

[B40] Bahrman N, Gouy A, Devienne-Barret F, Hirel B, Vedele F, Le Gouis J (2005). Differential change in root protein pattern of two wheat varieties under high and low nitrogen nutrition levels. Plant Sci.

[B41] Foyer CH, Ferrario-Méry S, Noctor G, Lea PJ, Morot-Gaudry JF (2001). Interactions between carbon and nitrogen metabolism. Plant Nitrogen.

[B42] Sivasankar S, Rothstein S, Oaks A (1997). Regulation of the accumulation and reduction of nitrate by nitrogen and carbon metabolites in maize seedlings. Plant Physiol.

[B43] Klein D, Morcuende R, Stitt M, Krapp A (2000). Regulation of nitrate reductase expression in leaves by nitrate and nitrogen metabolism is completely overridden when sugars fall below a critical level. Plant Cell Environ.

[B44] Huppe HC, Turpin DH (1996). Appearance of novel glucose-6-phosphate dehydrogenase isoforms in Chlamydomonas reinhardtii during growth on nitrate. Plant Physiol.

[B45] Wang YH, Garvin DF, Kochian LV (2001). Nitrate-induce genes in tomato roots. Array analysis reveals novel genes that may play a role in nitrogen nutrition. Plant Physiol.

[B46] Li M, Villemur R, Hussey PJ, Silflow CD, Gantt JS, Snustad DP (1993). Differential expression of six glutamine synthetase genes in *Zea mays *. Plant Mol Biol.

[B47] Sakakibara H, Kawabata S, Hase T, Sugiyama T (1992). Differential effects of nitrate and light on the expression of glutamine synthetases and ferredoxin-dependent glutamate synthase in maize. Plant Cell Physiol.

[B48] Rockel P, Strube F, Rockel A, Wildt J, Kaiser WM (2002). Regulation of nitric oxide (NO) production by plant nitrate reductase *in vivo *and *in vitro*. J Exp Bot.

[B49] Igamberdiev AU, Bycova NV, Hill RD (2006). Nitric oxide scavenging by barley hemoglobin is facilitated by a monodehydroascorbate reductase-mediated ascorbate reduction of methemoglobin. Planta.

[B50] Lamattina L, Garcìa-Mata C, Pagnussat G (2003). Nitric oxide: the versatility of an extensive signal molecule. Annu Rev Plant Biol.

[B51] Stöhr C, Stremlau S (2006). Formation and possible roles of nitric oxide in plant roots. J Exp Bot.

[B52] Zhao DY, Tian QY, Li LH, Zhang WH (2007). Nitric oxide is involved in nitrate-induced inhibition of root elongation in *Zea mays *. Ann Bot.

[B53] Peschke VM, Sachs MM (1994). Characterization and expression of transcripts induced by oxygen deprivation in maize (*Zea mays *L.). Plant Physiol.

[B54] Igamberdiev AU, Hill RD (2004). Nitrate NO and haemoglobin in plant adaptation to hypoxia: an alternative to classic fermentation pathways. J Exp Bot.

[B55] Fritz C, Palacios-Rojas N, Fell R, Stitt M (2006). Regulation of secondary metabolism by the carbon-nitrogen status in tobacco: nitrate inhibits large sectors of phenylpropanoid metabolism. Plant J.

[B56] Kingston-Smith AH, Bollard AL, Minchin FR (2005). Stress-induced changes in protease composition are determined by nitrogen supply in non-nodulating white clover. J Exp Bot.

[B57] Simões I, Faro C (2004). Structure and function of plant aspartic proteinases. Eur J Biochem.

[B58] Askura T, Watanabe H, Abe K, Arai S (1995). Rice aspartic proteinases, oryzasin, expressed during seed ripening and germination, has a gene organization distinct from those of animal and microbial aspartic proteinases. Eur J Biochem.

[B59] Raven JA (1986). Biochemical disposal of excess H^+ ^in growing plants?. New Phytol.

[B60] Sakano K (1998). Revision of biochemical pH-stat: involvement of alternative pathway metabolisms. Plant Cell Physiol.

[B61] Britto DT, Kronzucker HJ (2005). Nitrogen acquisition, PEP carboxylase, and cellular pH homeostasis: new views on old paradigms. Plant Cell Environ.

[B62] Uhrig RG, She YM, Leach CA, Plaxton WC (2008). Regulatory monoubiquitination of phosphoenolpyruvate carboxylase in germinating castor oil seeds. JBC.

[B63] de Vetten NC, Ferl RJ (1994). Two genes encoding GF14 (14-3-3) proteins in *Zea mays*. Structure, expression, and potential regulation by G-box-binding complex. Plant Physiol.

[B64] Bihn EA, Paul AL, Wang SW, Erdos GW, Ferl RJ (1997). Localization of 14-3-3 proteins in the nuclei of Arabidopsis and maize. Plant J.

[B65] Roberts MR (2000). Regulatory 14-3-3 protein-protein interactions in plant cells. Curr Opin Plant Biol.

[B66] Bachmann M, Huber JL, Athwal GS, Wu K, Ferl RJ, Huber SC (1996). 14-3-3 proteins associate with the regulatory phosphorylation site of spinach leaf nitrate reductase in an isoform-specific manner and reduce dephosphorylation of Ser-543 by endogenous protein phosphatases. FEBS Lett.

[B67] Ikeda Y, Koizumi N, Kusano T, Sano H (2000). Specific binding of a 14-3-3 protein to autophosphorylated WPK4, an SNF1-related wheat protein kinase, and to WPK-4-phosphorylated nitrate reductase. JBC.

[B68] Dickson R, Weiss C, Howard RJ, Alldrick SP, Ellis RJ, Lorimer G, Azem A, Viitenen PV (2000). Reconstitution of higher plant chloroplast chaperonin 60 tetradecamers active in protein folding. JBC.

[B69] Averill RH, Bailey-Serres J, Kruger NJ (1998). Co-operation between cytosolic and plastidic oxidative pentose phosphate pathways revealed by 6-phosphogluconate dehydrogenase-deficient genotypes of maize. Plant J.

[B70] Malkin R, Niyogi K, Buchanan B, Gruissem W, Jones R (2000). Photosynthesis. Biochemistry and Molecular Biology of Plants.

[B71] Rapala-Kozik M, Kowalaska E, Ostrowska K (2008). Modulation of thiamine metabolism in *Zea mays *seedlings under conditions of abiotic stress. J Exp Bot.

[B72] Edwards GE, Franceschi VR, Voznesenskaya EV (2004). Single-cell C_4 _phothosynthesis versus the dual-cell (Kranz) paradigm. Annu Rev Plant Biol.

[B73] Ueno Y, Imanari E, Emura J, Yoshizawa-Kumagaye K, Nakajiama K, Inami K, Shiba T, Sakakibara H, Sugiyama T, Izui K (2000). Immunological analysis of the phosphorylation state of maize C4-form phosphoenolpyruvate carboxylase with specific antibodies raised against a synthetic phosphorylated peptide. Plant J.

[B74] Izui K, Matsumura H, Furumoto T, Kai Y (2004). Phosphoenolpyruvate carboxylase: a new era of structural biology. Annu Rev Plant Biol.

[B75] Nemchenko A, Kunze S, Feussner I, Kolomietes M (2006). Duplicate maize 13-lipoxygenase genes are differentially regulated by circadian rhythm, cold stress, wounding, pathogen infection, and hormonal treatments. J Exp Bot.

[B76] Feussner I, Bachmann A, Höhne M, Kindl H (1998). All three acyl moieties of trilinolein are efficiently oxygenated by recombinant His-tagged lipid body lipoxygenase *in vitro *. FEBS Lett.

[B77] James HE, Robinson C (1991). Nucleotide sequence of cDNA encoding the precursor of the 23 kDa protein of the photosynthetic oxygen-evolving complex from wheat. Plant Mol Biol.

[B78] Yoshiba Y, Yamaguchi-Shinozaki K, Shinozaki K, Harada Y (1995). Characterization of a cDNA clone encoding 23 kDa polypeptide of the oxygen-evolving complex of photosystem II in rice. Plant Cell Physiol.

[B79] Sourosa M, Aro EM (2007). Expression, assembly and auxiliary functions of photosystem II oxygen-evolving proteins in higher plants. Photosynth Res.

[B80] Ifuku K, Yamamoto Y, Ono T, Ishihara S, Sato F (2005). PsbP protein, but not PsbQ protein, is essential for the regulation and stabilization of photosystem II in higher plants. Plant Physiol.

[B81] Gaude N, Bréhélin C, Tischendorf G, Kessler F, Dörmann P (2007). Nitrogen deficiency in Arabidopsis affects galactolipid composition and gene expression and results in accumulation of fatty acid phytyl esters. Plant J.

[B82] Sakurai I, Mizusawa N, Wada H, Sato N (2007). Digalactosyldiacylglycerol is required for stabilization of the oxygen-evolving complex in photosystem II. Plant Physiol.

[B83] Cataldo DA, Haroon M, Schrader LE, Youngs VL (1975). Rapid colorimetric determination of nitrate in plant tissue by nitration of salicylic acid. Commun Soil Sci Plant Anal.

[B84] Ferrario-Méry S, Valadier MH, Foyer CH (1998). Overexpression of nitrate reductase in tobacco delays drought-induced decreases in nitrate reductase activity and mRNA. Plant Physiol.

[B85] Nelson NA (1944). A photometric adaptation of the Somogy method for the determination of glucose. JBC.

[B86] Moore S, Stein WH (1954). A modified ninhydrin reagent for the photometric determination of amino acids and related compounds. JBC.

[B87] Martínez-Garcia JF, Monte E, Quall PH (1999). A simple, rapid and quantitative method for preparing Arabidopsis protein extracts for immunoblot analysis. Plant J.

[B88] Lichtenthaler HK (1987). Chlorophylls and carotenoids: Pigments of photosynthetic biomembranes. Met Enzymol.

[B89] Heat RL, Packer K (1968). Photoperoxidation in isolated chloroplasts. I. Kinetics and stoichiometry of fatty acid peroxidation. Arch Biochem Biophys.

[B90] Krause GH, Weis E (1991). Chlorophyll fluorescence and photosynthesis: the basics. Annu Rev Plant Physiol Plant Mol Biol.

[B91] Genty B, Briantais JM, Baker NR (1989). The relationship between the quantum yield of photosynthetic electron transport and quenching of chlorophyll fluorescence. BBA.

[B92] Hurkman WJ, Tanaka CK (1986). Solubilization of plant membrane proteins for analysis by two-dimensional gel electrophoresis. Plant Physiol.

[B93] Laemmli UK (1970). Cleavage of structural proteins during the assembly of the head of bacteriophage. T4. Nature.

[B94] Neuhoff V, Arold N, Taube D, Ehrhardt W (1988). Improved staining of proteins in polyacrylamide gels including isoelectric focusing gels with clear background at nanogram sensitivity using Coomassie Brilliant Blue G-250 and R-250. Electrophoresis.

[B95] Magni C, Scarafoni A, Herndl A, Sessa F, Prinsi B, Espen L, Duranti M (2007). Combined electrophoretic approaches for the study of white lupin mature seed storage proteome. Phytochemistry.

[B96] National Center for Biotechnology Information. http://www.ncbi.nlm.nih.gov/.

[B97] Eng JK, McCormack AL, Yates JR (1994). An approach to correlate tandem mass spectral data of peptides with amino acid sequences in a protein database. J Am Soc Mass Spectrom.

[B98] Mackey AJ, Haystead TAJ, Pearson WR (2002). Getting more from less: algorithms for rapid protein identification with multiple short peptide sequences. Mol Cell Proteomics.

[B99] ExPASy Proteomics Server. http://www.expasy.org/.

