# Supplementary material for: Evaluation of protein pattern changes in roots and leaves of Zea mays plants in response to nitrate availability by two-dimensional gel electrophoresis analysis
Source: BMC Plant Biol. 2009 Aug 23;9:113. doi: 10.1186/1471-2229-9-113 (PMC2744680; doi:10.1186/1471-2229-9-113)
Supplement: Additional file 1 — Pictures of the plants. File shows the pictures of the experimental plant material at the different sampling times. [file 1471-2229-9-113-S1.pdf]

**Additional File 1**
**Pictures of the experimental plant material at the different sampling times**

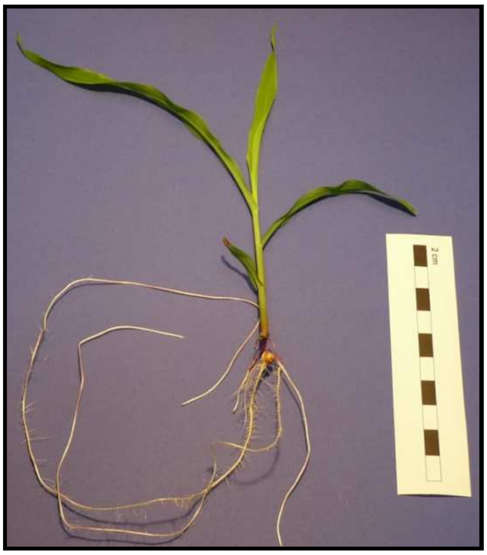

**$T_0$: plants grown for 17 days in the absence of nitrogen**

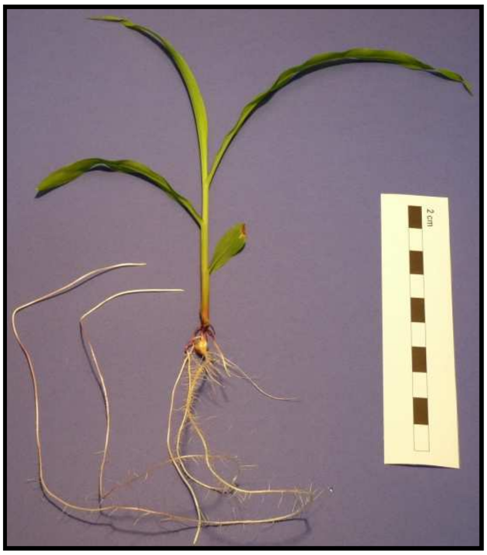

**$Ct_6$: plants kept for the further 6 h in the absence of nitrogen**

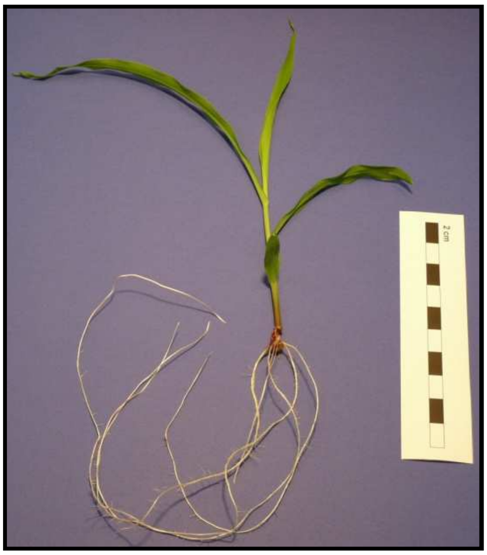

**$Nt_6$: plants grown for the last 6 h in the presence of 10 mM $NO_3^-$**

$Ct_{30}$: plants kept for the further 30 h in the absence of nitrogen

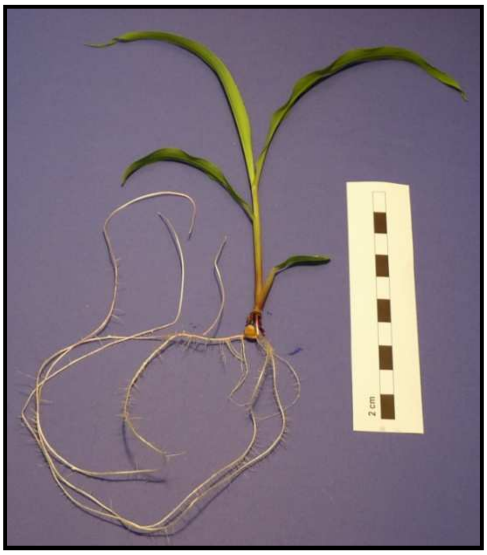

Detail of the shoot

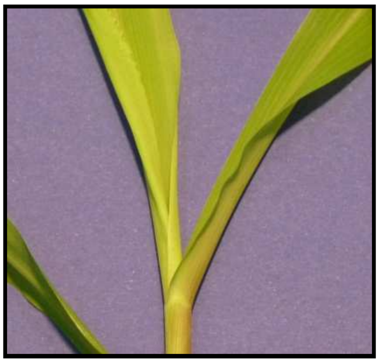

$Nt_{30}$: plants grown for the last 30 h in the presence of 10 mM $NO_3^-$

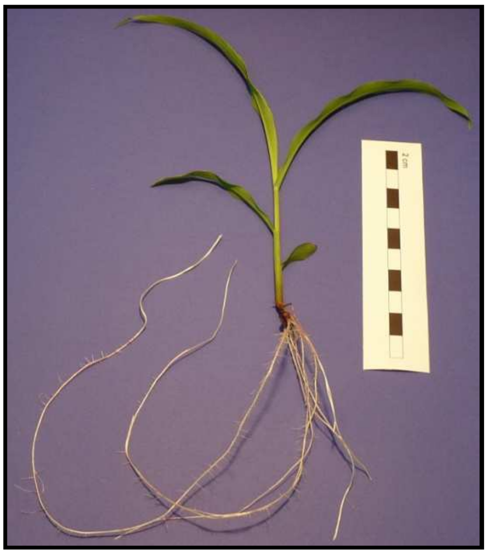

Detail of the shoot

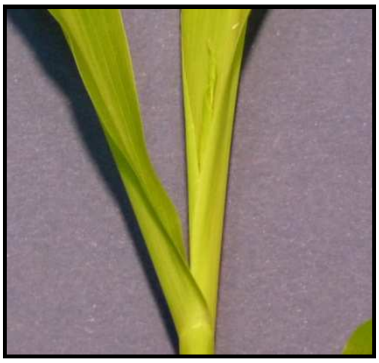

**$Ct_{54}$: plants kept for the further 54 h in the absence of nitrogen**

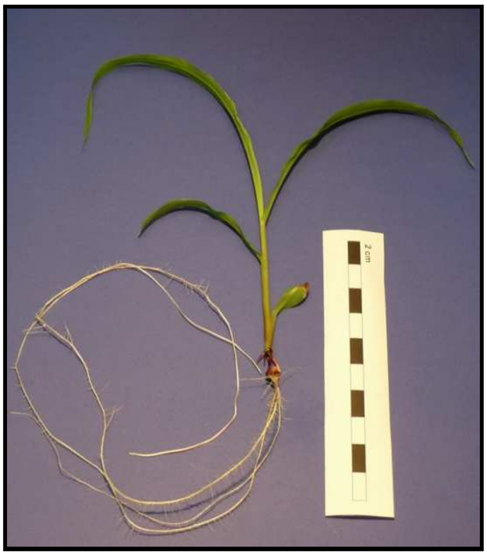

**Detail of the shoot**

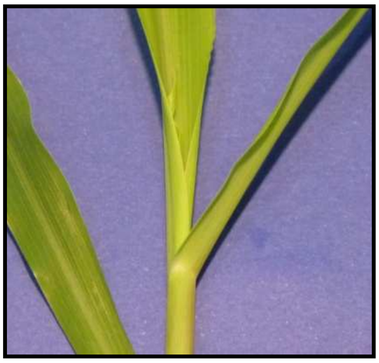

**$Nt_{54}$: plants grown for the last 54 h in the presence of 10 mM $NO_3^-$**

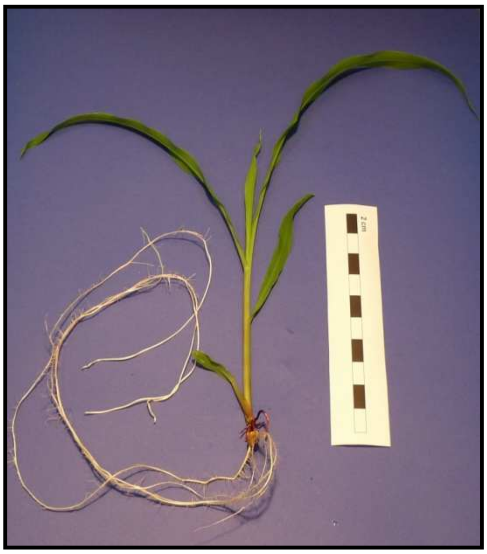

**Detail of the shoot**

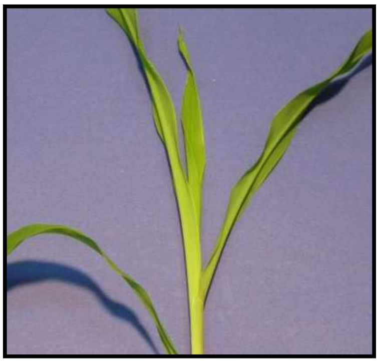

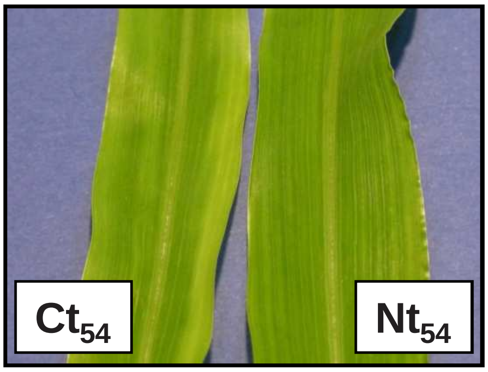


**Comparison between $Ct_{54}$ and $Nt_{54}$ leaf blades**
