# Supplementary material for: Evaluation of protein pattern changes in roots and leaves of Zea mays plants in response to nitrate availability by two-dimensional gel electrophoresis analysis
Source: BMC Plant Biol. 2009 Aug 23;9:113. doi: 10.1186/1471-2229-9-113 (PMC2744680; doi:10.1186/1471-2229-9-113)
Supplement: Additional file 2 — Caption of Additional file 3. Caption and legend of Additional file 3. [file 1471-2229-9-113-S2.doc]

**Caption of Additional file 3**

**List of the identified proteins by LC-ESI-MS/MS and bioinformatics analyses.** The table shows the sequence of all the peptides identified by MS/MS and the associated statistical information obtained from database searches conducted by BioworksBrowser using TurboSEQUEST® software. For each identified protein, statistical information related to direct protein database search or to alignment analysis of identified peptides by FASTS software are reported. **R/ :**spot of the root pattern. **L/ :**spot of the leaf pattern**. Spot ID**: spot identifier number. **Protein A.N.**: protein NCBI accession number (version). **DB**: databases downloaded from NCBI; NR: protein non-redundant database; NRm: subset of *Zea mays* protein; EST: subset of *Zea mays* EST. **n. pep**.: number of peptides used to identify the protein. **a.a. cov. (%)**: sequence coverage %. **Sf (pro):** protein SEQUEST Sf score. **FASTS (*E*) value**: FASTS expectation (*E*) values of the entry resulting from the alignment of peptides against NCBI NR non-redundant database. **Hom. Protein A.N.**: homologous protein NCBI accession number (version). **EST A. N.**: EST NCBI accession number (version). **Peptide**: sequence of the identified peptide; the symbol M* indicates oxidized methionine. **MH+**: molecular mass of the peptide; **z**: charge state of the peptide**. Sf (pep):** SEQUEST Sf score of the peptide. **Xcorr**: SEQUEST cross-correlation value. **ΔCn**: delta correlation value. **Sp**: SEQUEST preliminary score. **(a):** values referred to the mature form of the protein.
