# Supplementary material for: Evaluation of protein pattern changes in roots and leaves of Zea mays plants in response to nitrate availability by two-dimensional gel electrophoresis analysis
Source: BMC Plant Biol. 2009 Aug 23;9:113. doi: 10.1186/1471-2229-9-113 (PMC2744680; doi:10.1186/1471-2229-9-113)
Supplement: Additional file 4 — Details of the protein sequences assigned to spot 53. File shows in detail the sequences of the PEPCase and UB proteins that were identified analyzing the spot 53 by LC-ESI-MS/MS, as well as the sequence alignment analysis to verify the presence of the domain involved in monoubiquitination of the enzyme. [file 1471-2229-9-113-S4.doc]

**Additional file 4 – Details of the protein assignment of spot 53**

**Sequence of phosphoenolpyruvate carboxylase (PEPCase) identified by LC-ESI-MS/MS analysis. The peptides identified (see Additional file 3) are reported in red.**

**>gi|3132310|dbj|BAA28170.1| phosphoenolpyruvate carboxylase [Zea mays]**

MPERHQSIDAQLRLLAPGKVSEDDK**LVEYDALLVDR**FLDILQDLHGPHLREFVQECYELSAEYENDRDEARLGELGSKLTSLPPGDSIVVASSFSHMLNLANLAEEVQIAHRRRIKLKRGDFADEASAPTESDIEETLKRLVSQLGKSREEVFDALK**NQTVDLVFTAHPTQSVR**RSLLQKHGRIRNCLRQLYAKDITADDKQELDEALQREIQAAFRTDEIRR**TPPTPQDEMR**AGMSYFHETIWKGVPKFLRRIDTALKNIGINERLPYNAPLIQFSSWMGGDRDGNPRVTPEVTRDVCLLARMMAANLYFSQIEDLMFELSMWRCSDELRIRADELHRSSRKAAKHYIEFWKQVPPNEPYRVILGDVRDKLYYTRERSRHLLTSGISEILEEATFTNVEQFLEPLELCYRSLCACGDKPIADGSLLDFLRQVSTFGLALVKLDIRQESDRHTDVLDSITTHLGIGSYAEWSEEKRQDWLLSELRGKRPLFGSDLPQTEETADVLGTFHVLAELPADCFGAYIISMATAPSDVLAVELLQRECHVKHPLRVVPLFEKLADLEAAPAAVARLFSIDWYMDRINGKQEVMIGYSDSGKDAGRLSAAWQMYKAQEELIKVAKHYGVKLTMFHGRGGTVGR**GGGPTHLAILSQPPDTIHGSLR**VTVQGEVIEHSFGEELLCFRTLQRYTAATLEHGMHPPISPKPEWRALMDEMAVVATKEYRSIVFQEPRFVEYFRSATPETEYGRMNIGSRPSKRKPSGGIESLRAIPWIFAWTQTRFHLPVWLGFGAAIKHIMQKDIRNIHILREMYNEWPFFRVTLDLLEMVFAK**GDPGIAAVYDK**LLVADDLQSFGEQLRKNYEETKELLLQVAGHKDVLEGDPYLKQRLRLRESYITTLNVCQAYTLKR**IRDPSFQVSPQPPLSK**EFTDESQPAELVQLNQQSEYAPGLEDTLILTMKGIAAGMQNTG

**Sequence of ubiquitin (UB) identified by LC-ESI-MS/MS analysis. The peptide identified (see Additional file 1) is reported in red.**

**gi|59800418|sp|P69319.1|UBIQ_MAIZE RecName: Full = Ubiquitin Swiss-Prot reviewed**

MQIFVKTLTGKTITLEVESSDTIDNVKAKIQDKEGIPPDQQRLIFAGKQLEDGR**TLADYNIQK**ESTLHLVLRLRGG

**Amino acid sequence alignment of BAA28170.1 with ABR29876.1 (PEPCase of germination castor oil seeds, Uhrig et al., 2008) to verify the presence of the domain involved in the monoubiquitination.**

**ABR29876.1** 7 EKLASIDAQLRLLVPAKVSEDDKLVEYDALLLDRFLDILQDLHGEDLKETVQECYELSAE 66

E+ SIDAQLRLL P KVSEDDKLVEYDALL+DRFLDILQDLHG L+E VQECYELSAE

**BAA28170.1** 3 ERHQSIDAQLRLLAPGKVSEDDKLVEYDALLVDRFLDILQDLHGPHLREFVQECYELSAE 62

**ABR29876.1** 67 YEGKHDPRKLDELGNLLTSLDPGDSIVIAKSFSHMLNLANLAEEVQIAYRRRNKLKKGDF 126

YE D +L ELG+ LTSL PGDSIV+A SFSHMLNLANLAEEVQIA+RRR KLK+GDF

**BAA28170.1** 63 YENDRDEARLGELGSKLTSLPPGDSIVVASSFSHMLNLANLAEEVQIAHRRRIKLKRGDF 122

**ABR29876.1** 127 ADENSATTESDIEETFKRLVIDLKKSPEEVFDALKNQTVDLVLTAHPTQSIRRSLLQKHA 186

ADE SA TESDIEET KRLV L KS EEVFDALKNQTVDLV TAHPTQS+RRSLLQKH

**BAA28170.1** 123 ADEASAPTESDIEETLKRLVSQLGKSREEVFDALKNQTVDLVFTAHPTQSVRRSLLQKHG 182

**ABR29876.1** 187 RIRNCLAQLYAKDITPDDKQELDEALQREIQAAFRTDEIRRTAPTPQDEMRAGMSYFHET 246

RIRNCL QLYAKDIT DDKQELDEALQREIQAAFRTDEIRRT PTPQDEMRAGMSYFHET

**BAA28170.1** 183 RIRNCLRQLYAKDITADDKQELDEALQREIQAAFRTDEIRRTPPTPQDEMRAGMSYFHET 242

**ABR29876.1** 247 IWKGVPKFLRRVDTALKNIGINERVPYNAPLIQFSSWMGGDRDGNPRVTPEVTRDVCLLA 306

IWKGVPKFLRR+DTALKNIGINER+PYNAPLIQFSSWMGGDRDGNPRVTPEVTRDVCLLA

**BAA28170.1** 243 IWKGVPKFLRRIDTALKNIGINERLPYNAPLIQFSSWMGGDRDGNPRVTPEVTRDVCLLA 302

**ABR29876.1** 307 RMMAANLYYSQIEDLMFELSMWRCSDELRVRADELHRSSKRDSKHYIEFWKQVPPSEPYR 366

RMMAANLY+SQIEDLMFELSMWRCSDELR+RADELHRSS++ +KHYIEFWKQVPP+EPYR

**BAA28170.1** 303 RMMAANLYFSQIEDLMFELSMWRCSDELRIRADELHRSSRKAAKHYIEFWKQVPPNEPYR 362

**ABR29876.1** 367 VILGDLRDKLYQTRERSRQMLSHGNSDIPEEATFTNVEQFLEPLELCYRSLCSCGDQPIA 426

VILGD+RDKLY TRERSR +L+ G S+I EEATFTNVEQFLEPLELCYRSLC+CGD+PIA

**BAA28170.1** 363 VILGDVRDKLYYTRERSRHLLTSGISEILEEATFTNVEQFLEPLELCYRSLCACGDKPIA 422

**ABR29876.1** 427 DGSLLDFLRQVSTFGFSLVRLDIRQESDRHTDVMDTITKHLEIGSYREWSEERRQEWLLS 486

DGSLLDFLRQVSTFG +LV+LDIRQESDRHTDV+D+IT HL IGSY EWSEE+RQ+WLLS

**BAA28170.1** 423 DGSLLDFLRQVSTFGLALVKLDIRQESDRHTDVLDSITTHLGIGSYAEWSEEKRQDWLLS 482

**ABR29876.1** 487 ELSGKRPLFGPDLQRTDEVADVLDTFHVIAELPADSFGAYIISMATAPSDVLAVELLQRE 546

EL GKRPLFG DL +T+E ADVL TFHV+AELPAD FGAYIISMATAPSDVLAVELLQRE

**BAA28170.1** 483 ELRGKRPLFGSDLPQTEETADVLGTFHVLAELPADCFGAYIISMATAPSDVLAVELLQRE 542

**ABR29876.1** 547 CHVKQPLRVVPLFEKLADLEAAPAALARLFSIDWYRNRINGKQEVMIGYSDSGKDAGRFS 606

CHVK PLRVVPLFEKLADLEAAPAA+ARLFSIDWY +RINGKQEVMIGYSDSGKDAGR S

**BAA28170.1** 543 CHVKHPLRVVPLFEKLADLEAAPAAVARLFSIDWYMDRINGKQEVMIGYSDSGKDAGRLS 602

**ABR29876.1** 607 AAWQLYKAQEELIKVAKQFGVK**L**TMFHGRGGTVGRGGGPTHLAILSQPPDTIHGSLRVTV 666

AAWQ+YKAQEELIKVAK +GVKLTMFHGRGGTVGRGGGPTHLAILSQPPDTIHGSLRVTV

**BAA28170.1** 603 AAWQMYKAQEELIKVAKHYGVKLTMFHGRGGTVGRGGGPTHLAILSQPPDTIHGSLRVTV 662

**ABR29876.1** 667 QGEVNEQSCGEEHLCFRTLQRFTAATLEHGMHPPVSPKPEWRKLMDEMAVIATEEYRSIV 726

QGEV E S GEE LCFRTLQR+TAATLEHGMHPP+SPKPEWR LMDEMAV+AT+EYRSIV

**BAA28170.1** 663 QGEVIEHSFGEELLCFRTLQRYTAATLEHGMHPPISPKPEWRALMDEMAVVATKEYRSIV 722

**ABR29876.1** 727 FKEPRFVEYFRLATPELEYGRMNIGSRPSKRKPSGGIESLRAIPWIFAWTQTRFHLPVWL 786

F+EPRFVEYFR ATPE EYGRMNIGSRPSKRKPSGGIESLRAIPWIFAWTQTRFHLPVWL

**BAA28170.1** 723 FQEPRFVEYFRSATPETEYGRMNIGSRPSKRKPSGGIESLRAIPWIFAWTQTRFHLPVWL 782

**ABR29876.1** 787 GFGAAFKHVIQKDVRNLHMLQEMYNEWPFFRVTIDLVEMVFAKGDPGIAALYDKLLVSQD 846

GFGAA KH++QKD+RN+H+L+EMYNEWPFFRVT+DL+EMVFAKGDPGIAA+YDKLLV+ D

**BAA28170.1** 783 GFGAAIKHIMQKDIRNIHILREMYNEWPFFRVTLDLLEMVFAKGDPGIAAVYDKLLVADD 842

**ABR29876.1** 847 LWSFGERLRTNYEETKRLLLQIAGHKDLLEGDPYLKQRLRLRDSYITTLNVCQAYTLKRI 906

L SFGE+LR NYEETK LLLQ+AGHKD+LEGDPYLKQRLRLR+SYITTLNVCQAYTLKRI

**BAA28170.1** 843 LQSFGEQLRKNYEETKELLLQVAGHKDVLEGDPYLKQRLRLRESYITTLNVCQAYTLKRI 902

**ABR29876.1** 907 RDPNYNVTLRPHISKEIMESSKPADELVKLNPKSDYAPGLEDTLILTMKGVAAGLQNTG 965

RDP++ V+ +P +SKE + S+PA ELV+LN +S+YAPGLEDTLILTMKG+AAG+QNTG

**BAA28170.1** 903 RDPSFQVSPQPPLSKEFTDESQPA-ELVQLNQQSEYAPGLEDTLILTMKGIAAGMQNTG 960

Uhrig RG, She YM, Leach CA, Plaxton WC: **Regulatory monoubiquitination of phosphoenolpyruvate carboxylase in germinating castor oil seeds**. *JBC* 2008, **283:**29650-29657.
